# Global changes in electricity consumption during COVID-19

**DOI:** 10.1016/j.isci.2021.103568

**Published:** 2021-12-03

**Authors:** Elizabeth Buechler, Siobhan Powell, Tao Sun, Nicolas Astier, Chad Zanocco, Jose Bolorinos, June Flora, Hilary Boudet, Ram Rajagopal

**Affiliations:** 1 Mechanical Engineering, Stanford University, Stanford, CA 94305, USA; 2 Civil and Environmental Engineering, Stanford University, Stanford, CA 94305, USA; 3 Economics, Stanford University, Stanford, CA 94305, USA; 4 School of Public Policy, Oregon State University, Corvallis, OR 97331, USA

**Keywords:** Energy resources, Energy policy, Energy sustainability, Energy systems

## Abstract

Understanding how the COVID-19 pandemic has altered electricity consumption can provide insights into society's responses to future shocks and other extreme events. We quantify changes in electricity consumption in 58 different countries/regions around the world from January-October 2020 and examine how those changes relate to government restrictions, health outcomes, GDP, mobility metrics, and electricity sector characteristics in different countries. We cluster the timeseries of electricity consumption changes to identify impact groupings that capture systematic differences in timing, depth of initial changes, and recovery rate, revealing substantial heterogeneity. Results show that stricter government restrictions and larger decreases in mobility (particularly retail and recreation) are most tightly linked to decreases in electricity consumption, although these relationships are strongest during the initial phase of the pandemic. We find indications that decreases in electricity consumption relate to pre-pandemic sensitivity to holidays, suggesting a new direction for future research.

## Highlights

• Electricity consumption changes during COVID-19 are estimated for 58 regions
• Impacts on electricity consumption are highly heterogeneous across regions
• Consumption changes are tightly linked to mobility and government restrictions
• Consumption changes also relate to a region's pre-pandemic sensitivity to holidays

## Graphical abstract

**Figure:**
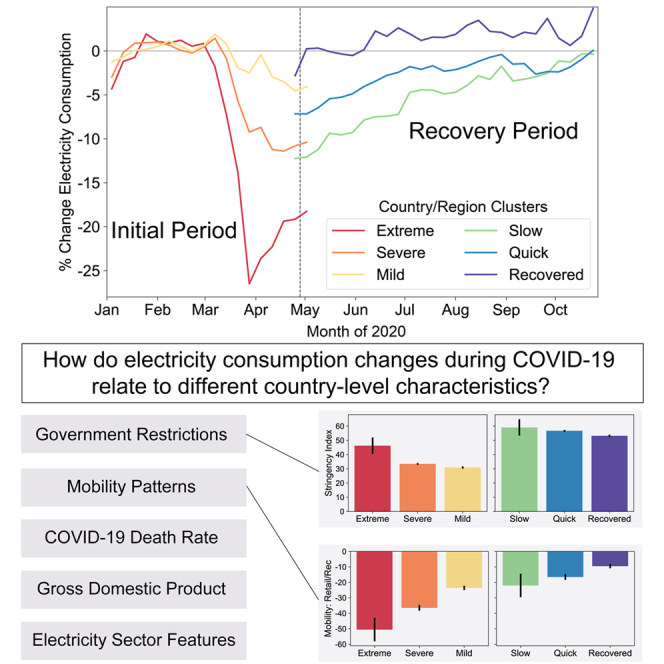

## Introduction

Efforts to stop the spread of COVID-19 have had profound impacts on daily life around the world. Self- and government-imposed restrictions have limited travel, social interaction, and in-person working and schooling and reduced commercial and industrial operations. During the height of the pandemic, as well as in areas that are still recovering, these changes to daily life have shifted where, when, and how electricity is consumed. Analyzing these impacts is important for several reasons.

First, analyzing the response to the COVID-19 pandemic allows for a better understanding of how electricity systems respond to large shocks. The pandemic and associated government and human responses have been unprecedented in nature. Yet, given the increased frequency of climate change-driven extreme weather events (Perera et al., 2020) and the possibility of other infrequent disruptive events such as pandemics and recessions, we can expect to see future shocks to electricity systems. Decreases and/or fluctuations in consumption like those caused by the COVID-19 pandemic can affect electricity grid operation, balancing, and forecasting (Elavarasan et al., 2020). Understanding and anticipating the impacts of shocks like COVID-19 on the electricity sector is critical to maintaining grid reliability and building resilience to adverse events. Many recent works have attempted to estimate the decrease in consumption caused by the pandemic (Agdas and Barooah, 2020; Alhajeri et al., 2020; Alkhraijah et al., 2021; Bahmanyar et al., 2020; Cicala, 2020a, 2020b; Edomah and Ndulue, 2020; EPIC, 2020; Eryilmaz et al., 2020; Halbrugge et al., 2021; Hauser et al., 2021; IEA, 2021; Jiang et al., 2021; Leach et al., 2020; Lopez Prol and O, 2020; McWilliams and Zachmann, 2020b, 2020a; Narajewski and Ziel, 2020; Percy and Mountain, 2020; Ruan et al., 2020; Santiago et al., 2021; Gillingham et al., 2020; Werth et al., 2020); however, they have been limited either by their timescale, geographic scope, modeling approach, and/or incomplete accounting of seasonal, weather, and temporal effects. Most other analyses are limited to individual countries or small groups of countries, particularly in the United States and Europe where high-resolution data are more readily available and only cover the initial stages of the pandemic. These significant differences in methodology make it challenging or impossible to conduct a meaningful meta-analysis of results from different studies. A detailed summary of the approaches in the existing literature is included in Table S4 in the supplemental information.

Second, analyzing how pandemic restrictions have impacted electricity consumption can reveal the potential power of governments and policy to change long-standing consumption patterns. Individual choices around mobility and policies such as stay at home orders have been proposed as drivers behind changes in electricity consumption (Lopez Prol and O, 2020; Percy and Mountain, 2020; Ruan et al., 2020; Werth et al., 2020). Yet, the stringency of government restrictions has varied significantly over time and between countries (Hale et al., 2020). Other factors, such as the local severity of the COVID-19 pandemic (Ruan et al., 2020) and the characteristics of a country's electricity system (Leach et al., 2020) (e.g., sectoral composition) have also been proposed as potentially being linked to electricity consumption changes. However, the relationship between changes in electricity consumption and this combination of factors has not been analyzed across a broad selection of countries over a long duration of the pandemic.

Third, some scholars (Cicala, 2020a; McWilliams and Zachmann, 2020a) have argued that electricity consumption could serve as a bellwether for both the economic impacts of and recovery from the pandemic and other crises because traditional economic measures (i.e., employment, gross domestic product or GDP) are collected over longer time horizons and thus more difficult to incorporate into daily decision-making. A better understanding of how different factors are related to changes in electricity consumption during the pandemic can inform its utility as an economic metric moving forward.

In this paper, we apply a unified approach to address the above challenges. First, we quantify the impact of the pandemic on electricity consumption worldwide during different phases of the pandemic, accounting for seasonal, weather, and temporal effects. Then, we analyze the relationship between changes in electricity consumption and various other proposed factors, including the severity of government restrictions, health outcomes, individual changes in mobility, change in GDP, and characteristics of each country's electricity system using panel regression. Understanding how these metrics relate to electricity consumption changes can help inform how electricity consumption may respond to future shocks. Finally, we characterize heterogeneity in the responses between countries by using clustering techniques to identify groups of countries with similar impacts based on the time-varying shapes of their responses. This allows us to identify connections between the severity and timing of a country's initial response to the shock and the shape of its recovery. We apply this framework to electricity consumption data from 58 countries and regions around the world, representing about 60% of the world's population and 75% of global electricity consumption (The World Bank, 2020a), from January to October 2020.

**Figure 1:**
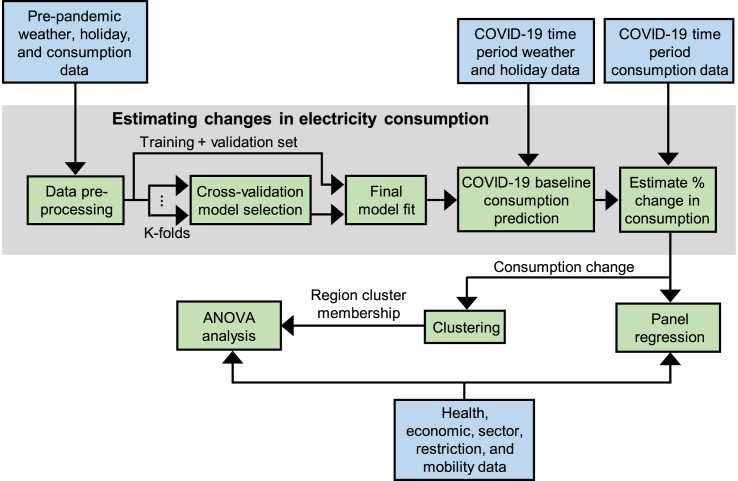
Overall analytic framework

Our estimates of electricity consumption impacts are obtained using country or region-specific regression models, which estimate what consumption would have looked like absent the pandemic (Figure 1), accounting for weather and temporal patterns in consumption. The difference between realized and baseline consumption provides an estimate of the observed changes in electricity consumption. This baselining approach to identifying event-driven consumption changes is common in the electricity utility sector, particularly for assessing the load impacts of demand response and peak pricing events (Chao, 2010; Couglin et al., 2009; Mathieu et al., 2011). It also accounts for several complex and potentially nonlinear effects of exogenous variables on consumption and provides flexibility to accommodate the heterogeneity in load behavior across countries and regions by using out-of-sample validation for model selection.

In the literature, papers have either used a simple comparison with historical consumption data (Alhajeri et al., 2020; Bahmanyar et al., 2020; Edomah and Ndulue, 2020; Halbrugge et al., 2021; Santiago et al., 2021; Werth et al., 2020), evaluated the effect of the pandemic on electricity consumption directly from coefficients of a regression model (Cicala, 2020a; Hauser et al., 2021; Leach et al., 2020; T. Gillingham et al., 2020), or used the baselining approach (Agdas and Barooah, 2020; Alhajeri et al., 2020; Lopez Prol and O, 2020; Narajewski and Ziel, 2020; Ruan et al., 2020). Many efforts to date account for weather in some way, fewer incorporate temporal effects (week, day, season, holidays), and even fewer incorporate any out-of-sample validation (Agdas and Barooah, 2020; Lopez Prol and O, 2020; Ruan et al., 2020).

Given the shortcomings in the literature outlined above, our work provides the most geographically comprehensive analysis to date of the relationship between the pandemic and electricity consumption across the globe. Our systematic approach, which uses interpretable models and is applied to a large geographic scope, allows us to observe patterns across countries and understand the variation in impacts during the pandemic. In the remainder of the paper, we describe our results on changes in electricity consumption across countries, the relationships between region/country-level factors and changes in electricity consumption, and our clustering analysis. Finally, we discuss the broader implications of our findings.

## Results

### Changes in electricity consumption at scale

Results show that, across the 53 countries and regions with daily electricity consumption data, total consumption declined by a daily average of 7.6% in April 2020, controlling for weather, seasonal, and temporal effects. Estimated daily reductions in electricity consumption range from the extreme (e.g., India's March to May reductions average 15%) to little impact at all (e.g., Australia's 2% average drop over the same period). Estimates of the impact on electricity consumption during the 2008 global financial crisis hover around 7% (IEA, 2020a), indicating that many of these COVID-related consumption reductions are unprecedented. The most drastic reductions in consumption occur in April, except in China where restrictions were most severe in February. Electricity consumption in nearly all regions recovered to near pre-pandemic levels by fall 2020.

**Figure 2:**
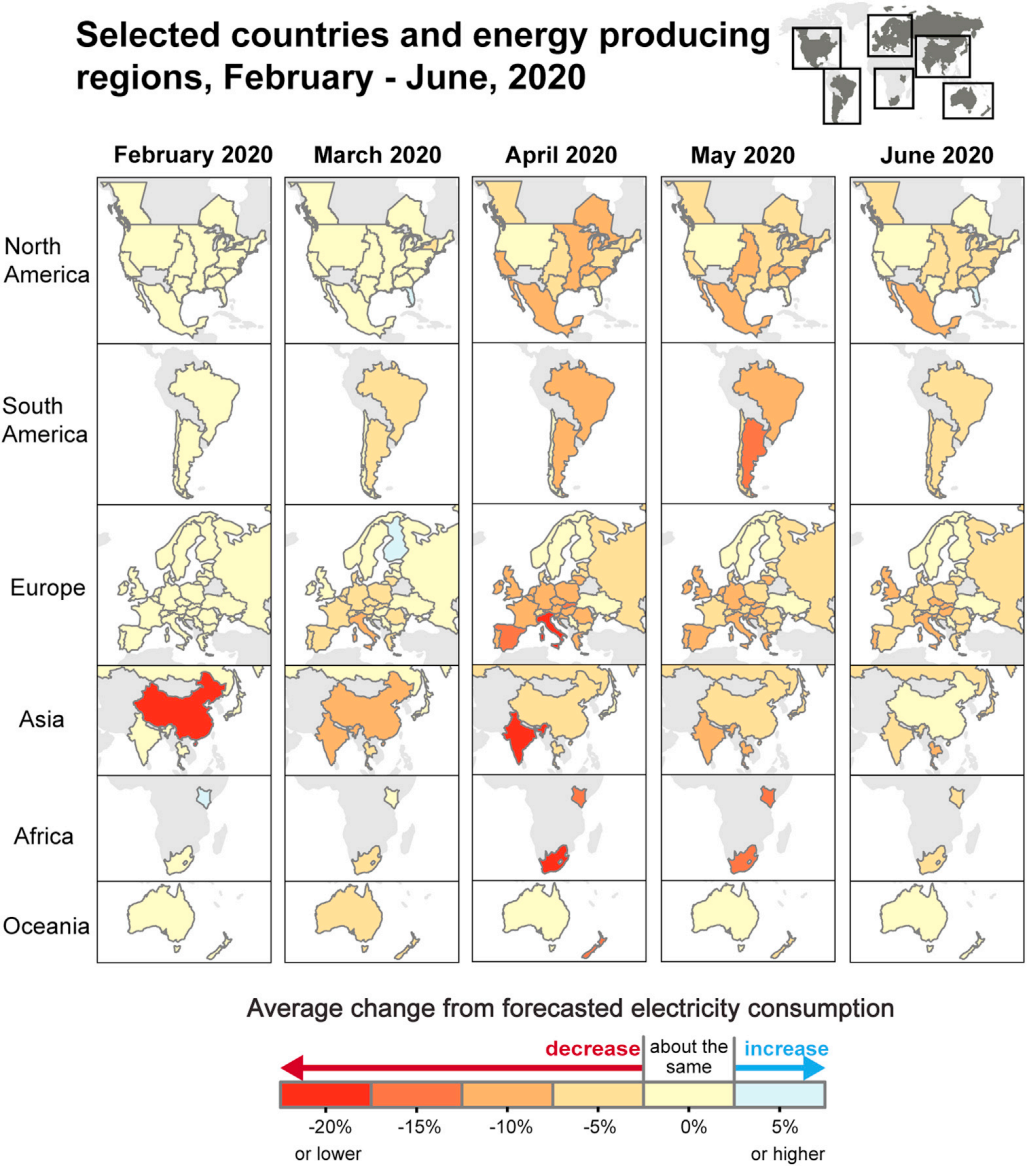
Estimated difference in modeled and actual electricity consumption for countries and regions across the world, Feb-Jun 2020 Color intensity corresponds to the change from modeled electricity consumption (+/− 2.5% per labeled midpoint in legend). Gray areas indicate missing data.

However, the impacts across countries are highly heterogeneous, with almost every continent having at least one country or region that experienced a dramatic reduction in consumption and one that did not (Figure 2). Several countries in Southern Europe (e.g., Italy, France, Spain) showed larger decreases in consumption, while little change occurred in Northern Europe (e.g., Sweden, Denmark, Finland). In Asia, both China and India experienced substantial decreases early in the pandemic, while Japan experienced a more muted change. For countries in South America (Argentina and Brazil), North America (Mexico), and Africa (South Africa), we also see examples of steep reductions, alongside countries with little impact (e.g., Chile).

**Figure 3:**
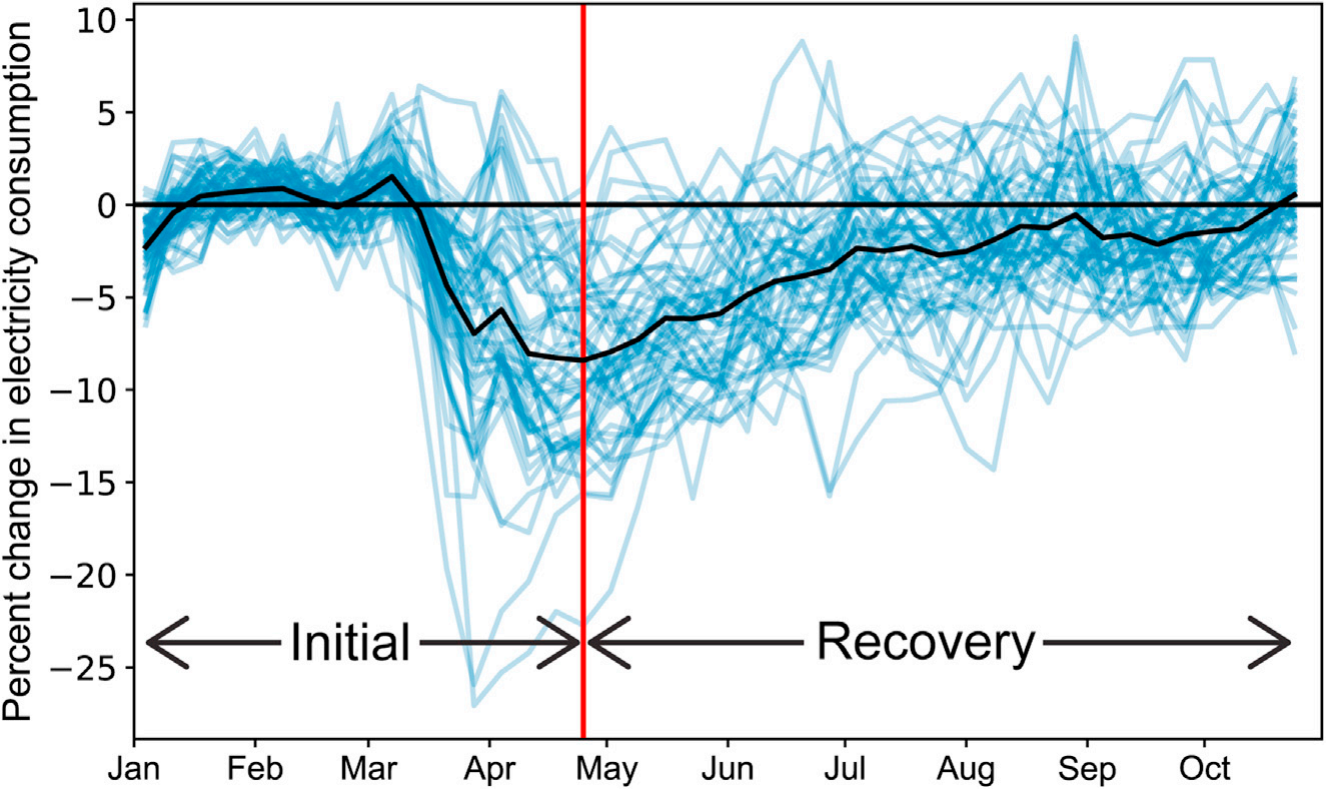
Percent change in weekly electricity consumption during COVID-19 pandemic for all countries over time, Jan-Oct 2020 The black line represents the average percent change in consumption across all countries. Note the change in late April from the *initial* period of decline to the later stage of *recovery*. January and February are included in the training and validation data and represent a portion of the pre-pandemic period.

Despite this heterogeneity, we observe an overall trend showing consumption declining in most countries in March and April and then starting to recover late April and early May. To consider how different factors relate to both the initial decline and recovery in electricity consumption across countries, we divide the analysis period into two phases (Figure 3): an *initial* phase (Jan-Apr 2020) and a *recovery* phase (May-Oct 2020). The timing of the two periods was identified using the breakpoints in a trend filtering analysis of the average change in consumption (see STAR Methods).

### Relationship between region/country-level factors and changes in electricity consumption

We next investigate which factors explain this observed heterogeneity in the responses among countries using guidance from the literature. We focus on factors that we and others have anticipated could have a relationship with changes in electricity consumption: COVID-19 health outcomes (Ruan et al., 2020), characteristics of the electricity sector, measures of the economy (Cicala, 2020a), government responses to the pandemic (Werth et al., 2020), and changes in different measures of human mobility (Ruan et al., 2020). For COVID-19 health outcomes, we analyze daily death rates in each region. One characteristic of the electricity sector we consider is the sectoral composition (commercial sector percentage). Various studies have observed decreased commercial and industrial consumption and increased residential consumption during the pandemic (Cicala, 2020b; Edomah and Ndulue, 2020; Santiago et al., 2021), so we might expect countries with a larger share of electricity consumption from industrial or commercial operations before the pandemic to have more precipitous declines in consumption during the pandemic. Another electricity sector characteristic we consider is the sensitivity of electricity consumption to holidays, measured from pre-pandemic data, which we call the “pre-pandemic holiday effect.” It is well known that electricity consumption is often lower on national, religious, and other cultural holidays, and as a result, special treatment is often given to these days in load forecasting (Brubacher and Wilson, 1976; Ziel, 2018). The pre-pandemic holiday effect considered in our study is a value equal to the mean fraction change in electricity consumption on a holiday, which is estimated from the coefficients of the electricity consumption regression model and is reflective of how changes in labor conditions affect electricity consumption. As a measure of economic activity, we use quarterly change in GDP. Government restrictions and responses to the pandemic are measured using the Oxford Stringency Index (Hale et al., 2020), a measure ranging from 1 to 100 cataloging the severity of “lockdown-style” policies designed to restrict disease transmission. Previous research has identified a relationship between higher levels of lockdown stringency and decreases in electricity consumption at the country level (Werth et al., 2020), and our expectation is that this relationship will persist across our sample of countries/regions. We measure mobility in terms of daily changes to retail and recreation destinations, a factor found in other research to be related to change in electricity consumption (Percy and Mountain, 2020; Ruan et al., 2020). Our selected factors include both time-varying and time-invariant characteristics.

To understand broadly how these different factors relate to changes in electricity consumption, we ran a panel regression with region/country random effects (Table 1). We use a random effects model, as opposed to a fixed effects model, as it allows us to include time-invariant characteristics in our modeling specifications. However, a model with region/country fixed effects produces similar results for time-varying factors (Table S3). Since both the change in electricity consumption and holiday effect are derived from the first-stage regression model, including the holiday effect in the panel regression could potentially create an endogeneity issue. Therefore, we do not include the holiday effect in the panel regression and instead analyze the correlation between electricity consumption changes and the holiday effect in a separate analysis.

**Table 1:** Results from panel regression analysis with region/country random effects for the initial and recovery periods

Variables	Model A		Model B	
	Initial period^a^		Recovery period	
	Estimate^b^	p Value	Estimate^b^	p Value
Oxford Stringency Index	−0.065∗∗∗	<0.001	−0.0780∗∗∗	<0.001
COVID-19 daily death rate (2-week average)	−1.576∗∗∗	<0.001	−0.1742	0.494
Change in daily mobility: retail and recreation	0.069∗∗∗	<0.001	0.0524∗∗∗	<0.001
Change in GDP (Q2 2020 vs. Q2 2019)	0.350∗∗∗	<0.001	0.0825	0.235
Commercial sector percentage	−0.346	0.924	−1.836	0.597
Intercept	6.2364∗∗∗	<0.001	4.0434∗∗	0.006
R-squared	0.4977		0.0921	
Adjusted R-squared	0.4969		0.0915	
Total sample size	3,233		8,760	
Groups	53		53	
Time intervals	61		124–169	

The dependent variable is percent change in daily electricity consumption.

a Owing to data availability, the initial period ranged from February 15 to April 28.

b Significance levels: ∗<0.05; ∗∗<0.01; ∗∗∗<0.001.

For the initial period (Table 1: Model A), we find that higher scores on the Oxford Stringency Index, which indicate more severe government restrictions, are associated with decreased electricity consumption (*b* = −0.065; p < .001), and higher COVID-related deaths per capita are also associated with decreased electricity consumption (*b* = −1.576; p < .001). Change in GDP (Q2 2020 versus Q2 2019) is positively associated with electricity consumption (*b* = 0.350; p < .001), indicating, for example, that countries with lower economic productivity were related to decreased electricity consumption, and vice versa. However, the relationship with the commercial sector percentage of electricity consumption is not significant.

In the recovery period (Table 1: Model B), COVID-19 deaths and change in GDP are no longer statistically significant, whereas the effect of the Oxford Stringency Index and the change in daily mobility related to retail and recreation are similar to the initial period model results. Moreover, when comparing model fit across initial and recovery periods, we find that the model for the initial period (R^2^ = 0.50) explains more variation in electricity consumption change compared with the recovery period (R^2^ = 0.09). Such a finding demonstrates that the connection between these included factors and electricity consumption change may be stronger during the initial period and weaker during the recovery period, motivating further investigation of what drove each country/region's recovery response.

Although the relationship with commercial sector percentage of electricity consumption is not significant in either period, we did observe a strong positive correlation between each country's pre-pandemic sensitivity of consumption to holidays and their maximum change in electricity consumption during the pandemic (correlation coefficient = 0.60; p < 0.001). This relationship between the two metrics suggests that the typical electricity consumption decrease on a holiday might carry useful information on the sensitivity of electricity consumption to economic activity.

### Clustering analysis of electricity consumption changes

To understand the heterogeneity between countries in more detail, we looked for common patterns in the responses in each of the initial and recovery periods. Clustering is an unsupervised approach to finding groups of similar timeseries or other types of samples in datasets and has been used extensively both in the power systems literature (Chicco et al., 2006; Kwac et al., 2014; Rhodes et al., 2014) and general scientific literature (e.g., transportation [Jiang et al., 2012], biomedical applications [Wiwie et al., 2015]). In this paper we used one of the simplest clustering methods, K-means clustering, to group countries with similar timeseries of change in electricity consumption. By using the timeseries vector for each country or region, this method compares the response shapes, capturing timing, magnitude, and non-linearities in the profiles of electricity consumption change. By dynamically assigning included regions or countries to clusters of different sizes, this method can also identify small but distinct groups that stand out from larger response patterns.

The clustering yielded surprising results that raised questions about why certain countries or regions were or were not grouped together. We also applied quantile-based methods for grouping the regions and countries, which rely on summary statistics of the timeseries, but we found them to provide limited characterizations of the heterogeneity in responses (Figures S5–S9). In the initial period, the extreme response patterns of Italy and India stand out sharply from the other countries and regions in our dataset, but quantile analysis could not identify this small, distinct group, since the method is limited to equal-sized groupings. In the recovery period, no single summary statistic for recovery timing or speed could fully characterize the variety of non-linear response shapes for quantile analysis. As a result, we found that clustering analysis was able to reveal richer patterns that better characterized the trajectories and heterogeneity among countries.

**Figure 4:**
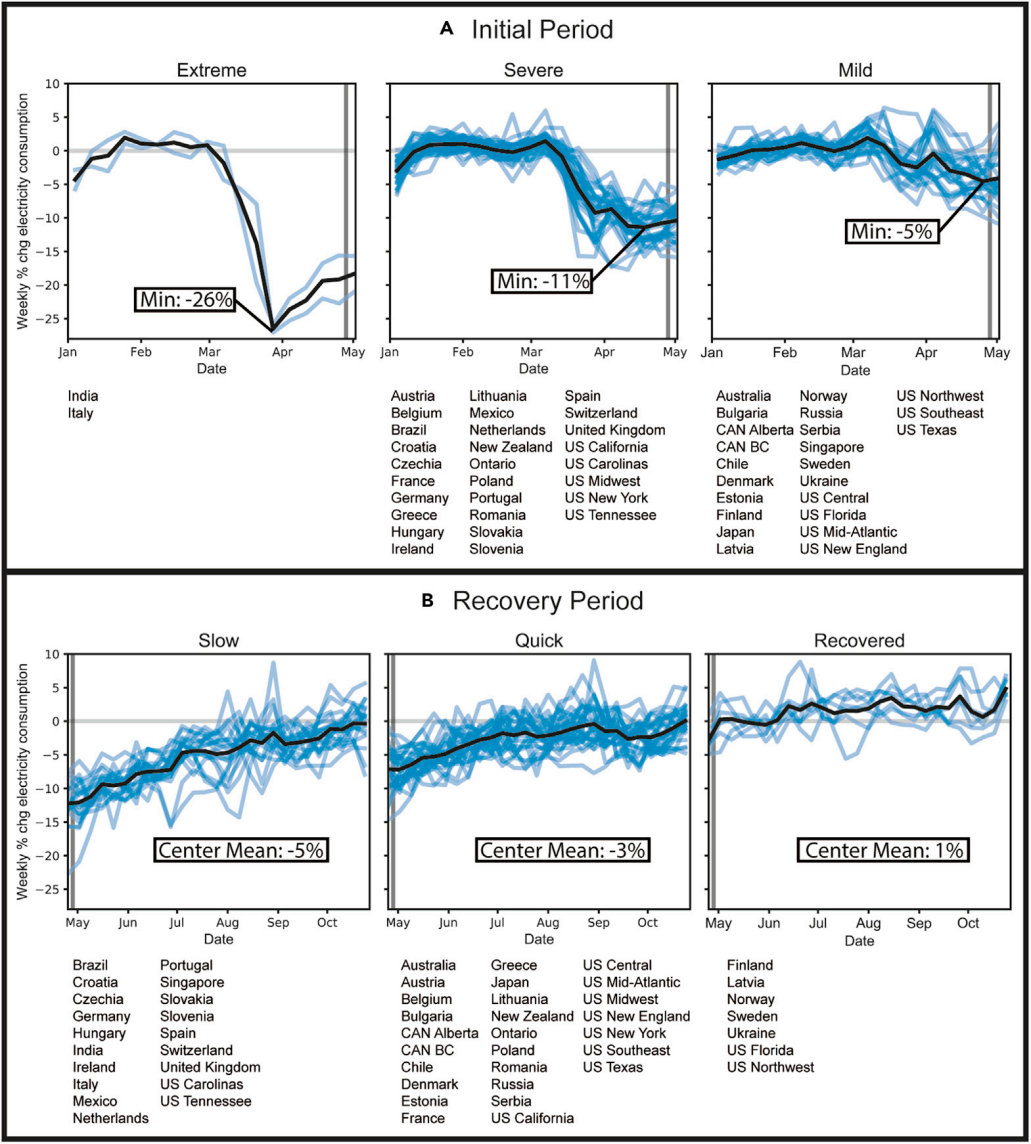
Electricity consumption impact groups during initial phase (Jan 1-Apr 28, 2020) and recovery phase (Apr 29-Oct 27, 2020) Impact clusters by the percent change in weekly electricity consumption for (A) the initial phase and (B) recovery phase. The plot shows the weekly data used for clustering, with each point located at the middle of its week. Black lines represent mean change in electricity use per cluster, the cluster center. Vertical dashed lines in the Initial period indicate when the cluster mean reached its minimum, with the value noted. January and February are included in the training and validation data and represent a portion of the pre-pandemic period.

For the initial period, our clustering produced three discrete impact groups, distinguished primarily by the timing and depth of the change (Figure 4A). The “Extreme” impact group, comprising only Italy and India, experienced the steepest change in weekly electricity consumption (averaging 26% at their lowest point in late March). The “Severe” impact group includes the most countries/regions in our sample (N = 28)—19 European countries, five US regions, New Zealand, Brazil, Mexico, and the Canadian Province of Ontario—and exhibited average electricity consumption reductions of as much as 11%. The “Mild” impact group (N = 23) had electricity consumption reductions averaging as much as 5%, generally occurring later in April, and includes seven US and two Canadian regions, nine European countries (particularly those in Scandinavia and Eastern Europe), Australia, Japan, Chile, Russia, and Singapore.

In the recovery period, countries clustered best into three discrete groups (Figure 4B), representing regions where consumption recovered (1) slowly, (2) quickly and then stayed near pre-pandemic levels after mid-July, or (3) stayed near pre-pandemic levels during the entire period (i.e., electricity consumption had already recovered). Slow recovering countries (N = 19) include both India and Italy from the Extreme group in the initial period, 16 regions/countries from the Severe group (12 from Europe, two US regions, Brazil, and Mexico), and one country from the Mild group (Singapore). Daily consumption change for the Slow recovering grouping is negative throughout the entire recovery period and only comes within 5% of baseline modeled consumption in the first week of July. Quick recovering regions/countries, which make up the largest recovery group (N = 27), include 12 countries from the Severe group and 15 from the Mild group, including the remaining eight US regions, all three Canadian regions, 11 European countries, Australia, New Zealand, Chile, Japan, and Russia. In contrast to the Slow recovering group, the daily consumption change for the Quick recovering grouping comes within 5% of baseline modeled consumption before the end of May. Only seven regions/countries make up the last group, where consumption levels stayed near pre-pandemic levels the entire period: Finland, Latvia, Norway, Sweden, Ukraine, and the US Northwest and Florida. All these countries/regions were in the Mild initial group, and some of them have been well known for their less restrictive responses to the pandemic (e.g., Sweden, Florida).

**Figure 5:**
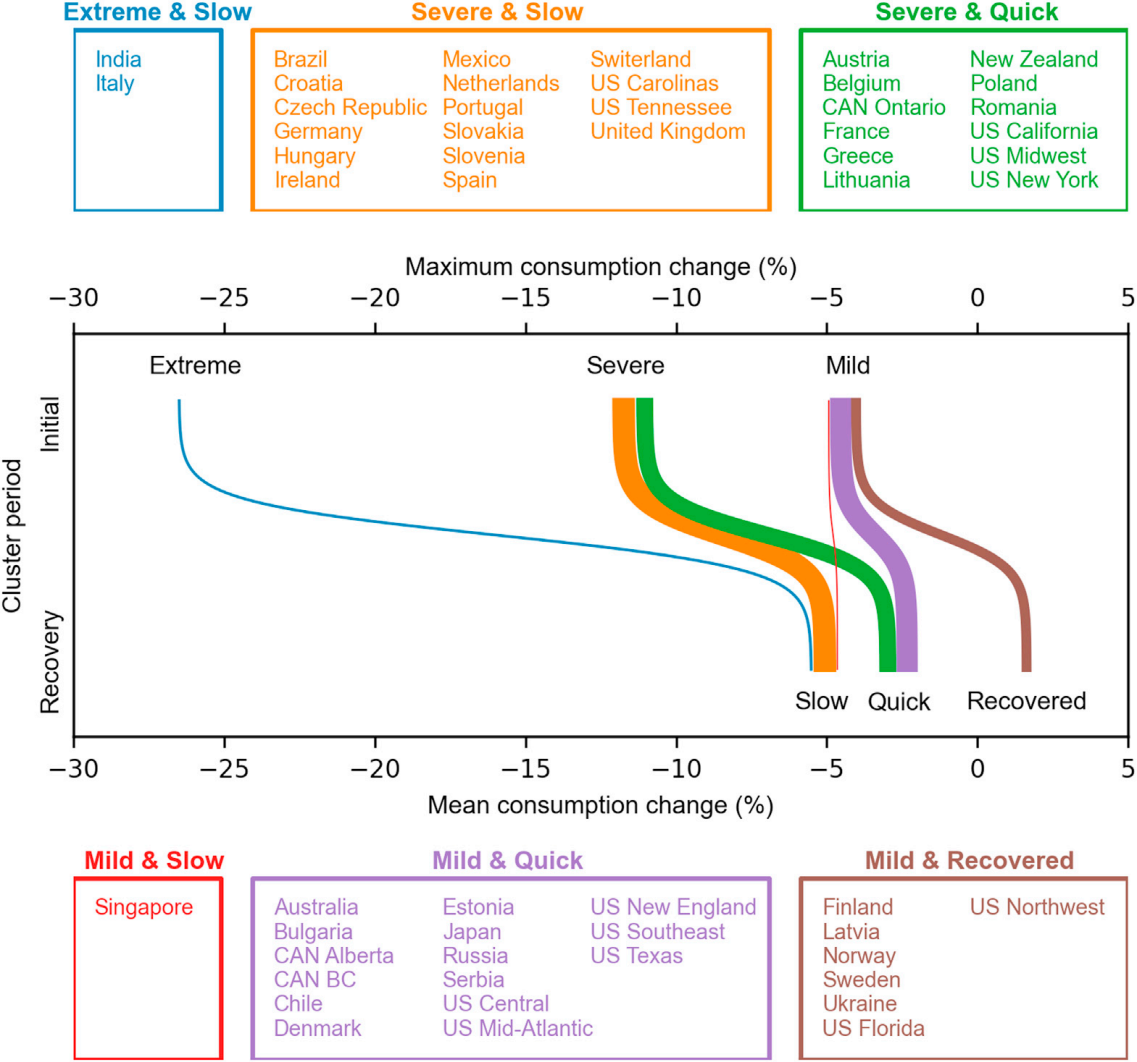
Country/region cluster membership for the initial analysis period (Jan-Apr) and recovery period (May-Oct) Top x axis shows the maximum percent change in weekly consumption for each cluster center during the initial period; bottom x axis shows the mean percent change in weekly consumption for each cluster center during the recovery period.

Therefore, in general, countries with larger initial decreases in consumption at the beginning of the pandemic took longer to recover (Figure 5). Both countries in the Extreme impact group in the initial period experienced Slow recoveries, and only countries in the Mild initial impact grouping were Already Recovered in the recovery period.

One-way ANOVAs were conducted to compare the relationships between region/country-level factors and our electricity consumption impact groupings in each period (see STAR Methods; Figure S10; Table S1). Post hoc comparisons were conducted using the Tukey HSD test (STAR Methods; Figure S10; Table S2). We do not find statistically significant differences across groups in the initial or recovery periods for sectoral composition and fatality rates, whereas we do find statistically significant differences between at least some groups for the change in second quarter GDP (between Q2 2019 and Q2 2020), mobility measures related to retail and recreation, and the Oxford Stringency Index in the initial period.

For the change in second quarter GDP, each group is statistically different from each other in the initial period with the mean of Extreme group lower than Severe and the mean of Severe group lower than Mild. There are also significant differences between some pairs of groups in the recovery period. However, for change in third quarter GDP, differences are not significant in either period. For the Oxford Stringency Index in the initial period, the Extreme group has the overall highest average stringency, which is statistically larger than the Mild group; however, differences are not significant in the recovery period. Human mobility measures broadly characterize individual-level decisions to travel or stay home and may or may not align with broader government restrictions. For many countries these measures were initially correlated with the Oxford Stringency Index, reflecting the strict early lockdowns, but became less strongly correlated to the index and more heterogeneous across countries and regions later in the pandemic period (Figure S4). We find statistically significant differences between our impact groups in terms of mobility related to retail and recreation, where the average value for countries/regions in the Extreme group is lower than in the Severe and Mild groups and the average value for countries/regions in the Severe is lower than in the Mild group (p = .033). In addition, for mobility related to grocery/pharmacy visits, there are differences across the groupings, with the Extreme group lower than the Mild group (p = .044).

## Discussion

Despite the pandemic's widespread impact around the world, our analysis of its impact on electricity consumption found that responses were highly heterogeneous. Each continent had both countries with extreme/severe and mild initial responses and quick and slow recoveries. Our observations on the relationship between electricity consumption changes and key factors, including COVID-19 deaths, government restrictions, changes in individual mobility, GDP, and characteristics of electricity systems, can help countries better understand and predict responses to future shocks. Some of our results are in line with previous work, whereas others are unexpected.

Government restrictions and mobility were significantly associated with changes in electricity consumption in both the initial and recovery time periods of the pandemic, which confirms that policies affecting individual behavior represent powerful tools to impact consumption. However, certain static variables associated with intrinsic properties of electricity systems also appear to be linked to changes in electricity consumption. Although sectoral composition was not a significant indicator for changes in electricity consumption during the pandemic, we did observe a correlation between electricity consumption changes and a country's pre-pandemic sensitivity of consumption to holidays.

The pre-pandemic holiday effect is an intrinsic property of an electricity system, measuring the average fraction of consumption that is typically shut down on a major holiday. This value likely captures hard-to-measure variables related to societal behavior, such as tendencies to modify energy-consuming behavior on holidays and the fraction of electricity tied to essential and non-essential work. It can also be estimated directly from historical consumption data without any bottom-up information about the characteristics of the electricity system. Our results suggest there might be a link between this metric and changes in consumption during the pandemic. However, since it is an intrinsic property of an electricity system, it can be difficult to disentangle and analyze independently of other factors. Future work should further analyze how this metric and other electricity sector properties could be used to help predict the impacts of future similar events and to understand the social and economic drivers of electricity consumption across different countries.

We found that the relationship between changes in electricity consumption and several time-varying factors tended to decouple in the recovery period of the pandemic. Changes in GDP were significantly associated with electricity consumption changes in the initial period but, surprisingly, were not significant in the recovery period. The link between electricity consumption and GDP was well established pre-pandemic (Baranzini et al., 2013; Lieskovsky, 2014; Megegaki, 2014), but this relationship may have evolved with sustained pandemic restrictions as behaviors and economic activity have adapted to this new situation. Given this result, electricity consumption may not be an accurate real-time measure for economic activity, until normal conditions return.

Similarly, for COVID-19 deaths, many countries experienced steep first waves in cases and deaths that mirrored their steep initial declines in electricity consumption in the initial period. In the recovery period, however, we found that COVID-19 deaths were no longer significantly associated with changes in electricity consumption. There were several notable exceptions in the initial period, such as India (Extreme impact group) and New Zealand (Severe impact group), which had very few cases yet very severe impacts on consumption during the initial period. These countries, instead, had strict government restrictions and abrupt changes in individual mobility during that time period, confirming the importance of policies. The lower R^2^ for the recovery period regression also indicates a much weaker relationship between electricity consumption and this set of factors, suggesting other factors not present during the initial period may be important to understanding the recovery.

One additional factor that we found did inform the recovery was the response of the country's electricity consumption to the initial shock. The pandemic was different from many previous shocks to the electricity system because it was not a single, short-term event affecting a specific geographic area. In many cases, structural changes made in the initial period persisted, like the shift to working from home. Our clustering analysis revealed the distinct, non-linear shapes of three typical response patterns, in each of the initial and recovery periods. Although these clusters did not group countries by geography, we found that a country's impact grouping in the initial period tended to relate to its grouping in the recovery period. In general, countries with more severe consumption impacts in the initial period took longer to recover, and no countries with Extreme or Severe initial impact shapes were included in the flat, Already Recovered impact group by the recovery period. For future consumption shocks, operators in each country or local region could use the severity of the initial response to help inform their prediction of the speed of recovery.

There are many implications of this research for both policy makers and electricity system operators as they anticipate future shocks to electricity consumption. There also remain open questions around the response during the COVID-19 pandemic and how the impacts we observed in this paper could relate to other factors. One potential extension would use our estimates of electricity consumption changes to improve calculations quantifying the global impact of the pandemic on carbon emissions. Recent studies (Bertram et al., 2021; Le Quere et al., 2020) aimed at assessing these impacts make simplifying assumptions about demand/consumption changes. Using our results could enable a more complete and detailed accounting of impacts at a global scale. To support such work, we are publishing our code and data for others to use (Buechler et al., 2021) (see key resources table). We believe this contribution will enable further and deeper exploration of the impacts of the pandemic and better preparation worldwide for future shocks to electricity consumption.

Further investigation into each country's electricity system is needed to characterize how country-level factors impacted the observed changes in electricity consumption and to understand why some countries with different characteristics, restrictions, and experiences with the pandemic clustered together. Although our analysis uncovered some of the drivers behind these clusters, other important questions remain about cluster groupings such as why, for example, the responses of Italy and India stood out so distinctly. Future work, therefore, should focus on understanding the mechanisms by which electricity consumption changes in response to disruptive or extreme events and drawing insights about the interplay between governmental, social, and economic factors and electricity systems.

### Limitations of the study

This study does not make causal claims about the relationships between changes in electricity consumption and our included factors. Based on our methodology, we only analyze how changes in electricity consumption are associated with our included factors.

## Supporting citations

The following references appear in the supplemental information: Benatia, 2020; Carroll et al., 2020; Ghiani et al., 2020; Kirli et al., 2021; Liu and Lin, 2021; de Mello Delgado et al., 2021.

## STAR★Methods

### Key resources table

**Table:** 

REAGENT or RESOURCE	SOURCE	IDENTIFIER
**Deposited data**		

All sources of data used in the study have been reported in the supplemental information (Table S5)		N/A
Data generated by this paper	This paper	https://doi.org/10.7910/DVN/EU6RCS

**Software and algorithms**		

RStudio	RStudio	https://cran.r-project.org/
Python 3.6.9	Python Software Foundation	https://www.python.org/downloads/
scikit-learn Python package	Pedregosa et al. (2011)	https://scikit-learn.org/stable/
statsmodels Python package	Seabold and Perktold (2010)	https://www.statsmodels.org/stable/index.html
plm R package	Croissant and Millo (2008)	https://cran.r-project.org/web/packages/plm/plm.pdf
lfe R package	Gaure (2013)	https://cran.r-project.org/web/packages/lfe/lfe.pdf
Code from this paper	This paper	https://doi.org/10.5281/zenodo.5794563

### Resource availability

#### Lead contact

Further information and requests for resources should be directed to and will be fulfilled by the lead contact, Ram Rajagopal (ramr@stanford.edu).

#### Materials availability

No materials were used in this study.

### Method details

#### Data sources

Sources of electricity data are listed in Table S5 in the supplemental information. Sub-hourly, hourly, or daily data were obtained for 53 of the countries and regions. Daily electricity consumption was calculated from these datasets for use in the regression model. Hourly electricity demand for the United States was obtained for 13 different subregions (EIA, 2020), which each represent an aggregation of multiple grid balancing authorities defined by the US Energy Information Agency (EIA). The Southwest region was left out of the analysis due to anomalous data/model behavior, leaving us with 12 subregions. Hourly or sub-hourly electricity data was obtained at the country level for most European countries (ENTSO-E, 2020). We excluded Cyprus, Montenegro, Luxembourg, and Bosnia from the analysis due to a combination of a high percentage of anomalous values in the demand data and high model validation errors, leaving us with 29 countries. The hourly data for Australia (AEMO, 2020) represents the electricity consumption for five states (New South Wales, Queensland, South Australia, Tasmania, and Victoria) which make up the majority (∼90%) of the country's population. Hourly data was obtained for three Canadian regions: Ontario (IESO, 2020), Alberta (AESO, 2020), and British Columbia (BC Hydro, 2020). Data for Japan (hourly) is limited to the TEPCO utility (TEPCO, 2020), which serves 35% of the population. Data for New Zealand (Electricity Authority, 2020), Russia (SO-CDU, 2020), Mexico (CENACE, 2020), Brazil (ONS, 2020), and Chile (Coordinador Electrico Nacional, 2020) are reported at the country-level with hourly resolution. Electricity data for India is reported at the country-level with daily resolution (POSOCO, 2020). Data for Singapore is reported at 30-minute intervals (Energy Market Authority, 2020). Daily consumption (MWh) was calculated from all of these datasets for use in the regression model. Small amounts of missing data for a small number of countries were padded for the clustering analysis. Additionally, there are five countries included in our study where only monthly electricity consumption data was available from public sources: China (China Electricity Council, 2020), South Africa (Open Data for Africa, 2020), Thailand (EPPO, 2020), Argentina (CAMMESA, 2020), and Kenya (KNBS, 2020).

Population-weighted daily heating degree day (HDD) and cooling degree day (CDD) data for US states and census regions were used in the models for the US regions (NOAA, 2020). Population-weighted HDD and CDD statistics were generated for all remaining regions using temperature data from weather stations in the ASOS Network (ISU, 2020) (located in cities with more than 100,000 inhabitants) and for which measurements were available over the full 2016-2020 period. We obtained a database of holiday dates for each country (Time and Date, 2020), and then manually selected which holidays were included in the model based on observations of the sensitivity of consumption to each holiday in the historical data.

For metrics of human mobility patterns, we used daily data from the Google COVID-19 Community Mobility Report (Google, 2020). This dataset includes changes in mobility related to workplaces, transit stations, grocery stores and pharmacies, residences, parks, and retail or recreational activities, and is available at the state or country level. Each metric is given as a percent change over a baseline from January 3rd to February 6th, 2020, accounting for the day of the week. Metrics for the 12 US regions were calculated from the state level data, weighted by population.

To measure the severity of COVID-19 restrictions we use the Stringency Index developed through the Oxford COVID-19 Government Response Tracker (OxCGRT) project (Hale et al., 2020). The index ranges in value from 0 to 100 based on the strictness of restrictions on people's behavior. Components of the index include the level of school, workplace and public transport closures, cancellation of public events, restrictions on gatherings, controls on international and internal movement, public information campaigns, and stay at home orders. Metrics for the 12 US regions were again calculated using state level data.

Health data including cases and deaths due to COVID-19 were obtained from the Johns Hopkins Coronavirus Resource Center (Johns Hopkins University, 2021). Quarterly changes in GDP for most countries were obtained through the OECD database (OECD, 2020), and for US states through the US Bureau of Economic Analysis (BEA, 2020a; 2020b). GDP data for a few remaining countries were obtained from several news articles and other sources (Budapest Business Journal, 2020; Business Times, 2020; Focus Economics, 2020a, 2020b, 2020c, 2020d, 2020e, 2020f; SeeNews, 2020; Total Croatia News, 2020). Population data was obtained from The World Bank (The World Bank, 2020b) and Statistics Canada (Statistics Canada, 2020). Sectoral decomposition of electricity consumption for each country as of 2018 was obtained from the International Energy Agency (IEA, 2020b). For Canada, the national values for the Stringency Index, GDP change, and sectoral decomposition were used for each of the 3 provinces.

#### Electricity consumption change estimation

To estimate changes in electricity consumption, we developed country or region-specific regression models for predicting baseline electricity consumption. Daily consumption changes were calculated by comparing the model estimate with actual consumption from January to October 2020. The regression model for predicting daily consumption for a given country/region is given by:

(Equation 1) $$y_{t}=\alpha_{r}+\overset{n_{w}}{\underset{k=1}{\sum }}\alpha_{h,k}h_{t}^{k}+\overset{n_{w}}{\underset{k=1}{\sum }}\alpha_{c,k}c_{t}^{k}+\overset{11}{\underset{i=1}{\sum }}\alpha_{m,i}m_{i,t}+\overset{6}{\underset{i=1}{\sum }}\alpha_{w,i}w_{i,t}+\overset{n_{y}-1}{\underset{i=1}{\sum }}\alpha_{a,i}a_{i,t}+\overset{n_{s}}{\underset{i=1}{\sum }}\alpha_{s,i}s_{i,t}+\varepsilon_{t}$$

where *y*_*t*_ is the daily consumption on day *t*, *h*_*t*_ is the number of daily heating degree days (HDD) of a given region on day *t*, *c*_*t*_ is the number of daily cooling degree days (CDD) of a given region on day t, $m_{i,t}$, $a_{i,t}$, $w_{i,t}$, and $s_{i,t}$ are dummy variables for the month of the year, year, day of the week, and holidays, and $\varepsilon_{t}$ is the error term. HDD and CDD values were calculated using a base temperature of 65°F. $n_{y}$ is equal to the number of years of data included in the training, validation, and test sets and $n_{s}$ is the number of different holidays included in the model. The yearly dummy variables account for long-term, consistent load growth patterns and the monthly dummy variables account for seasonal effects not captured by the HDD and CDD terms. Holiday dummy variables were also added for specific days of the year that typically experience reduced consumption but are not official national or religious holidays, such as the days between Christmas and New Year's Eve.

The parameters of the regression model were identified using ordinary least-squares regression using the statsmodels Python package (Seabold and Perktold, 2010). The order of the polynomial HDD and CDD terms $n_{w}$ (between 1 and 4) was selected based on the out-of-sample validation error using k-fold cross validation with 10 folds. For most regions, the 10 folds were randomly selected from data between January 2016 and the end of February 2020. For some regions, data was only available beginning in January 2017 or January 2018. Therefore, at least two years of data was used for training and validation for each region. Validation errors for each fold were defined as the root mean squared error (RMSE) of the predictions, expressed as a percent of the average daily consumption in the dataset. The average validation error over all folds of the 10-fold cross-validation was used for model selection. For most regions, $n_{w}=4$ was selected, indicating that higher order HDD and CDD terms are necessary to account for nonlinear temperature dependencies. These effects are typically neglected by other studies. The final regression model was fit using the combined dataset from all 10 folds.

Mean out-of-sample validation errors for each country range between 1.26 and 4.94% RMSE (Figure S1). Note that errors over longer periods than one day, such as over a week or month, are substantially smaller than the daily errors shown. Daily training and validation errors are similar for each region, indicating that negligible model overfitting occurred.

Additional variables not accounted for in the model, such as economic factors, irregular growth rates, behind-the-meter PV generation, and non-holiday events, may affect baseline consumption in specific regions, but to a much lesser degree than the weather, temporal, and seasonal variables included in the model. One example observed in our results is a small dip in the estimated consumption change in mid-August in some European counties, which may be due to many people going on vacation. In our analysis, we also considered more complex, nonlinear models, which can provide small improvements in baseline consumption estimation for particular countries. However, the simple regression model defined in Equation 1 provides good generalizability and accuracy over all regions and allows us to develop a unified modeling framework despite having heterogeneous consumption behaviors in different regions.

The pre-pandemic holiday effect was calculated from the holiday dummy coefficients in Equation 1. Specifically, the mean fraction change in consumption on a holiday for each country/region was calculated by taking the mean of the significant holiday coefficients (using a p value threshold of .05), weighted by the number of times each holiday appears in the training set, and normalizing by the mean daily consumption of the dataset.

For the five countries with only monthly electricity consumption data, our estimates rely on monthly observations from either January 2012 (South Africa, Thailand), 2015 (China) or 2017 (Argentina, Kenya) to December 2019. We fit a linear regression model with monthly dummy variables and make country-specific model adjustments based on observed historical patterns. First, for countries that experience substantial growth in their electricity consumption (China, Kenya, Thailand), we add a time trend linear in the number of months since the beginning of our dataset. Second, if a correlation between temperature and electricity consumption is observed in the dataset, we include either monthly CDD (Thailand) or both monthly CDD and HDD (Argentina, China and South Africa) regressors in the model. Finally, for China we include a dummy variable for the month of the year during which the Spring Festival occurred (either January or February) which was found to improve model performance. These monthly estimates are not as accurate as our estimates derived from daily data. Confidence intervals typically range from +/− 3% (South Africa, Thailand), +/−5% (Argentina, China) or +/−6% (Kenya).

#### Panel regression

Panel regression models with region/country random effects were estimated using available data in the initial and recovery periods.

(Equation 2) $$\Delta y_{r,t}=\;\beta_{o}o_{r,t}+\beta_{d}d_{r,t}+\beta_{q}q_{r,t}+\beta_{g}\text{Δ}g_{r}+\beta_{b}b_{r}+u_{r}+\delta_{r,t}$$

For region r on day t, the modeled dependent variable was percentage change in daily electricity consumption, $\text{Δ}y_{r,t}$; time-varying independent variables included the factors Oxford Stringency Index, $o_{r,t}$, two week rolling average of the COVID-19 daily death rate, $d_{r,t}$, and change in daily mobility: retail and recreation, $q_{r,t}$. For region r, time-invariant independent variables included the factors change in GDP (Q2 2020 vs. Q2 2019), $\text{Δ}g_{r}$, and commercial sector percentage, $b_{r}$. $u_{r}$ is the region-specific random effect and $\delta_{r,t}$ is the error term. Factors were selected for model inclusion using insights from previous literature as well as multicollinearity considerations. COVID-19 daily death rate was converted to a two-week rolling average when applied in this analysis. In addition to random effects models, as a robustness check we also ran models with region/county fixed effects. For comparable time-varying characteristics, we observed the same sign and similar coefficient magnitude and significance (Table S3). The models were run using the plm (Croissant and Millo, 2008) and lfe (Gaure, 2013) R packages.

#### Clustering

K-Means clustering was used to segment the regions based on weekly electricity impacts for the initial and recovery periods of the pandemic. Use of the Euclidean distance metric allows these clusters to capture similarities in both the timing and magnitude of the response. Clustering was performed using the Scikit-learn Python package (Pedregosa et al., 2011). To select the number of clusters, we consulted the Scree plot of K-Means cluster inertia shown in Figures S2 and S3, and selected k=3 for both the initial response (Jan-Apr) and recovery (May-Oct) periods. The final week of the initial period ended April 28 and the final week of the recovery period ended October 27. Our breakdown of consumption impacts into initial and recovery periods was motivated by an unsupervised trend filtering algorithm (Tibshirani and Saunders, 2005), which identified breakpoints in consumption-weighted average change in electricity consumption across sample countries/regions. Analyses run with 2-5 break points indicated an upswing in electricity consumption after April 28, 2020 which was chosen as the end of the initial period. The recovery period runs from April 29 to October 27, 2020. To ensure reproducibility, the clustering was run with 100 random initializations and the clustering with the lowest inertia chosen; that clustering was also the most frequent.

#### Relationships to region/country-level factors

One-way ANOVAs were conducted to compare the relationships between region/country-level factors and our electricity consumption impact groupings in each period (Figure S10; Table S2). Post hoc comparisons were conducted using the Tukey HSD test (Figure S10; Table S3). We included several time-varying indicators in these analyses: the Oxford Stringency Index, COVID-19 daily cases and deaths, and change in daily mobility (workplaces, residences, transit stations, grocery and pharmacy, retail and recreation, and parks). In addition, we included several time-invariant independent variables: change in GDP (Q2 2020 vs. Q2 2019 and Q3 2020 vs. Q3 2019) and percent sectoral composition (commercial, residential, industrial, transport, and other). These region/country factors were chosen for their relevance to electricity consumption (e.g., change in GDP, sectoral composition), or to COVID's societal impacts (e.g., Oxford Stringency Index, COVID-19 daily death rate, change in mobility).

## Data Availability

• The data generated by our analysis has been deposited in the Harvard Dataverse: https://doi.org/10.7910/DVN/EU6RCS. Raw data can be downloaded from the sources listed in Table S5. Processed model input data will be shared by the lead contact upon request. • All original code has been deposited in the Zenodo database: https://doi.org/10.5281/zenodo.5794563. • Any additional information required to reanalyze the data reported in this paper is available from the lead contact upon request.
