# Supplementary material for: Global changes in electricity consumption during COVID-19
Source: iScience. 2021 Dec 3;25(1):103568. doi: 10.1016/j.isci.2021.103568 (PMC8641442; doi:10.1016/j.isci.2021.103568)
Supplement: Document S1. Figures S1–S10 and Tables S1–S5 [file mmc1.pdf]

iScience, Volume *25*

Supplemental information

Global changes in electricity consumption during COVID-19

Elizabeth Buechler, Siobhan Powell, Tao Sun, Nicolas Astier, Chad Zanocco, Jose Bolorinos, June Flora, Hilary Boudet, and Ram Rajagopal

Figure S1. Mean in-sample training errors and out-of-sample validation errors for the electricity consumption regression model for predicting the daily consumption of each region.

Values represent the mean RMSE over all 10 folds of the 10-fold cross validation and are expressed as a percent of the mean daily consumption for each region. Related to STAR Methods.

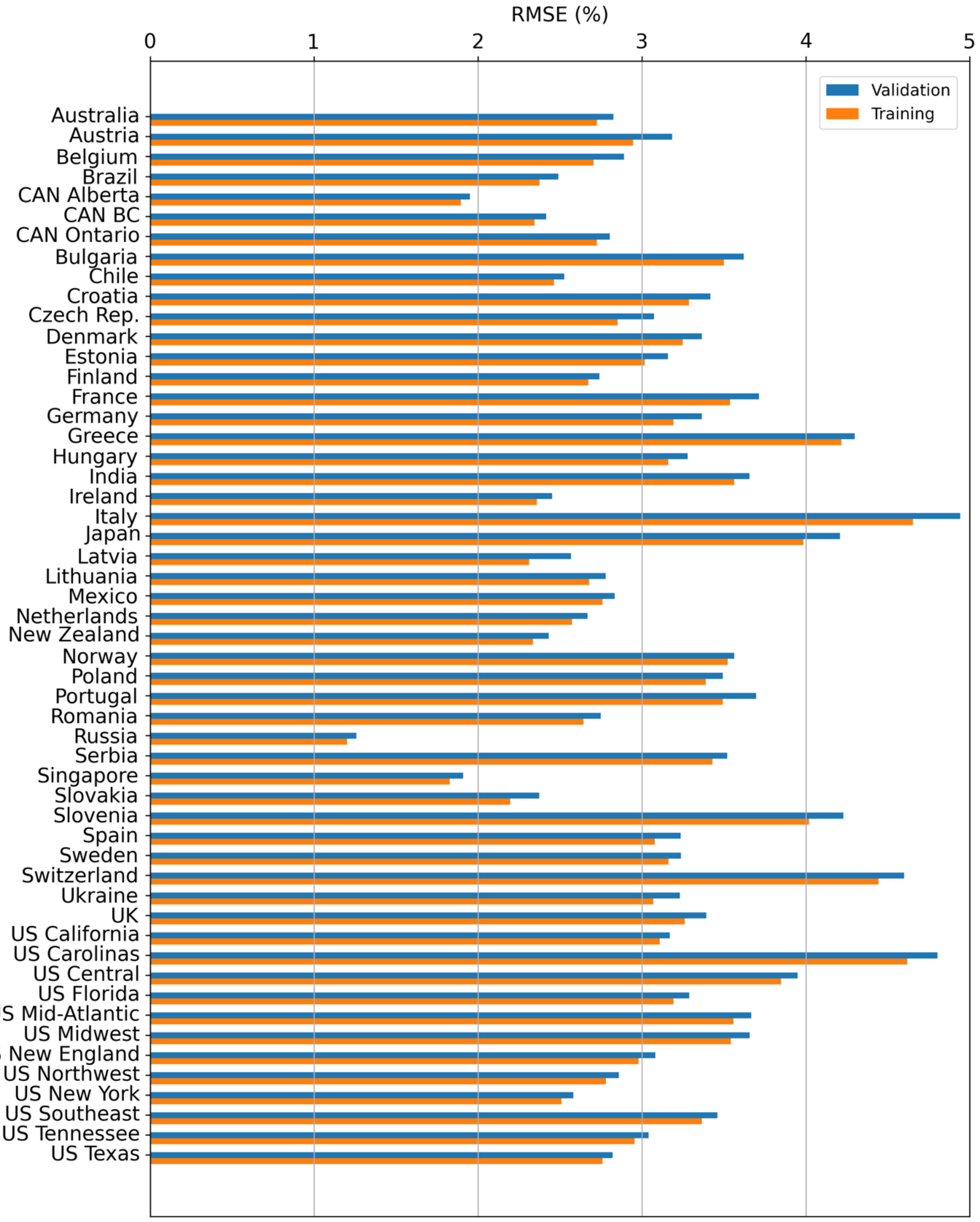

Figure S2. Elbow curve for K-means clustering of electricity consumption impact in initial period.

For each value of K, the model inertia is the sum of the squared distances between the samples and their closest cluster center. Related to STAR Methods.

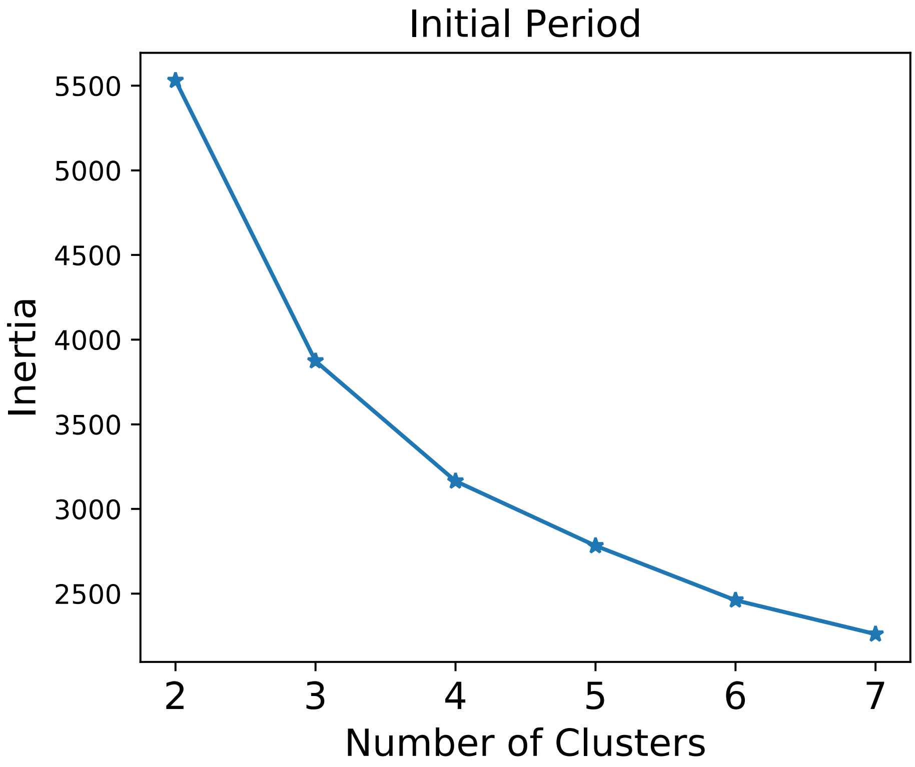

Figure S3. Elbow curve for K-means clustering of electricity consumption impact in recovery period.

For each value of K, the model inertia is the sum of the squared distances between the samples and their closest cluster center. Related to STAR Methods.

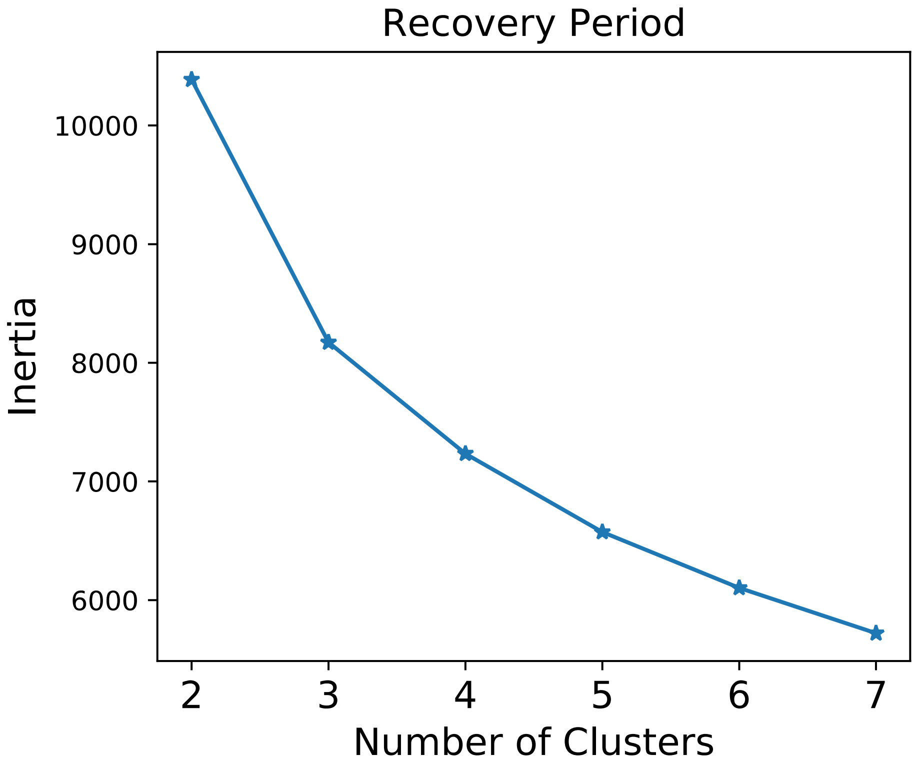

Figure S4. Correlation coefficients between mobility metrics and confinement stringency.

Countries' and regions' correlation coefficients showing the relationship between mobility metrics and confinement stringency during the two analysis periods. Related to STAR Methods.

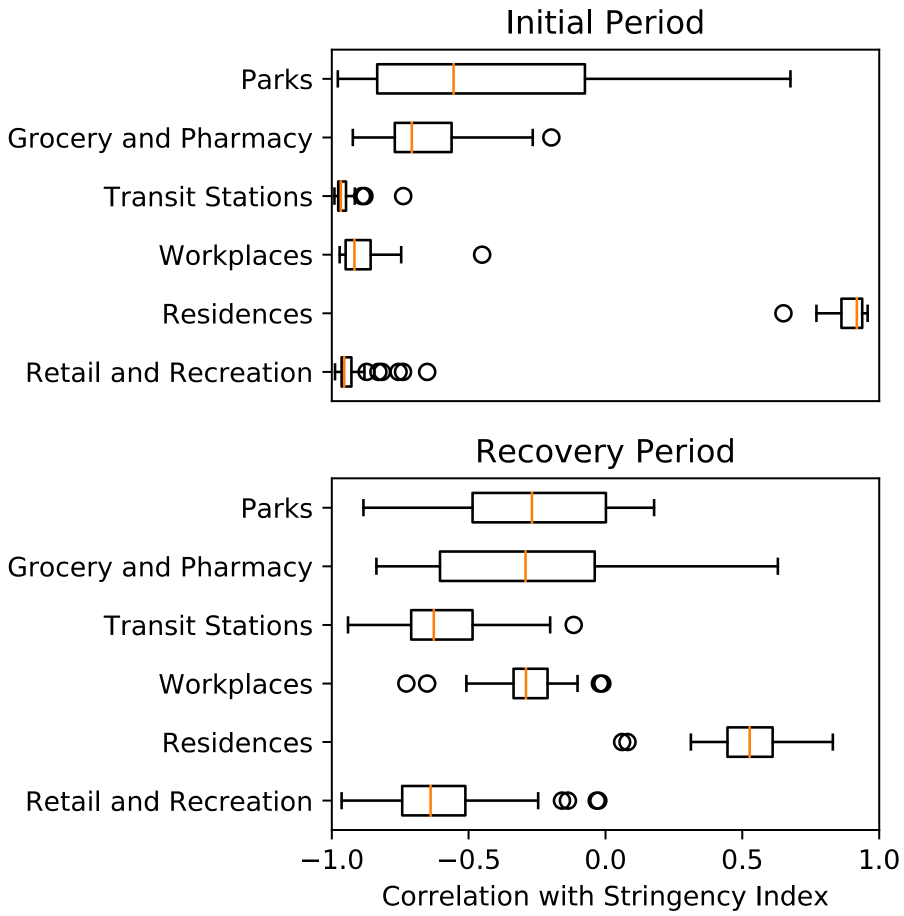

Figure S5: Comparison of K-means with tercile analysis in initial period.

The maximum drop during the initial period was used to group countries into terciles. In the two left most plots this metric is plotted for all countries/regions. The coloring showing the K-means and tercile groupings reveal significant differences between the two: the tercile analysis, limited to equal size clusters, cannot identify India and Italy as a distinct group. On the right timeseries plot: the top shows countries/regions grouped and colored by K-means cluster; the bottom shows countries/regions grouped by tercile, but still colored by K-means cluster. This shows the mismatch of shapes between these two methods, as well as highlighting how Italy and India standout in the initial period. Related to STAR Methods.

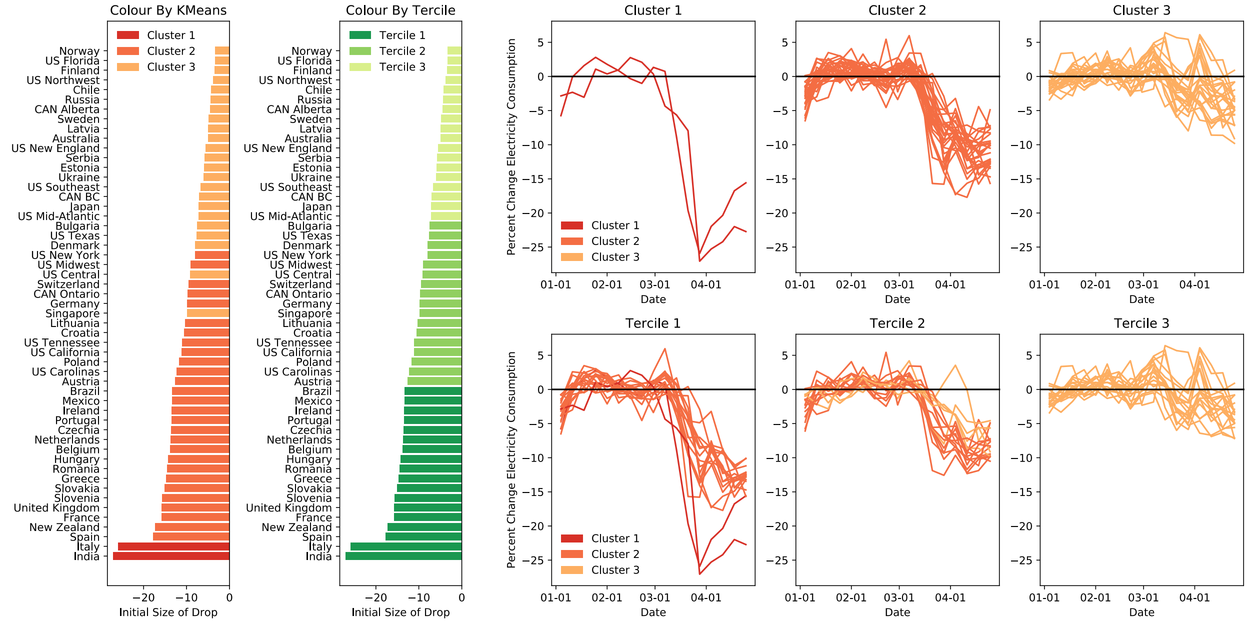

Figure S6: Comparison of K-means with quartile analysis in initial period.

The same as in Figure S5, the maximum drop during the initial period was used to group countries into quartiles. The number of clusters in the K-means analysis is similarly increased to 4 for comparison. The quartile method largely sorts the countries but is still unable to separate out the distinct cluster of India and Italy identified by the K-means algorithm. This difference persists as the number of clusters/groups is increased. Related to STAR Methods.

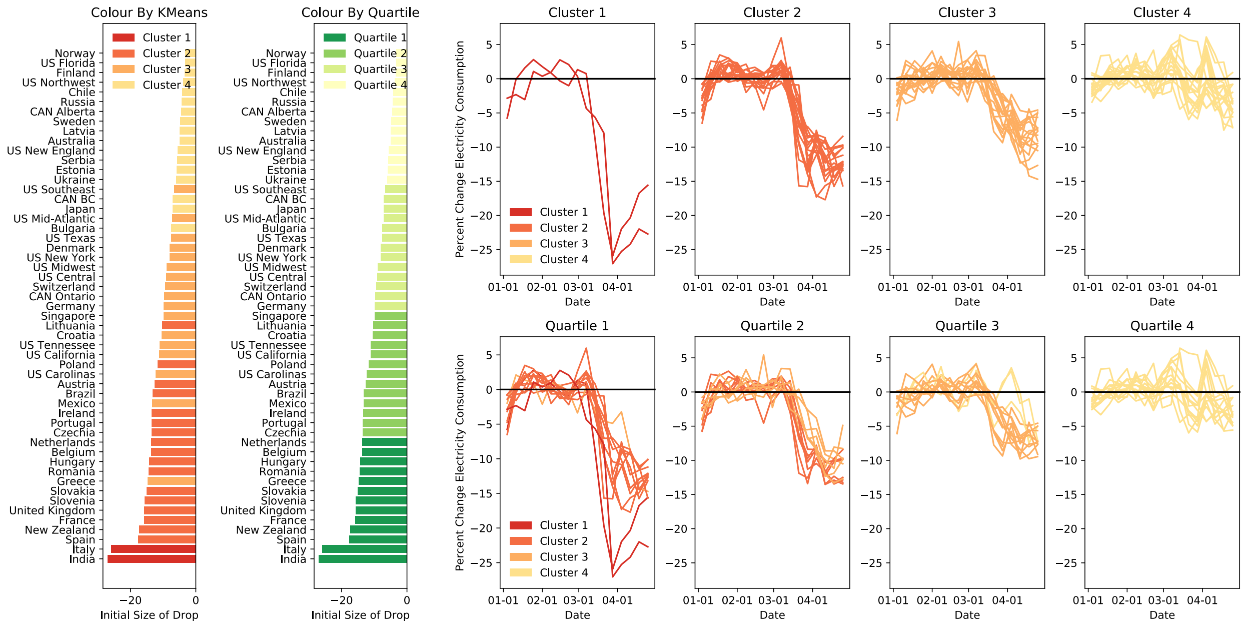

Figure S7: Comparison of K-means with tercile analysis in recovery period using zero cross.

The first week in the recovery period when each timeseries crosses zero was used here to group countries into terciles. In the two left most plots this metric is plotted for all countries/regions, and on the right the countries/regions are grouped either by their cluster (top) or tercile (bottom). This metric for recovery is very sensitive to noise, leading to significant differences between the results from terciles and K-means clustering as shown in the plot. For example, a small spike above zero caused by noise in the middle of a prolonged, slow recovery can misrepresent a country as having recovered more quickly. The K-means method avoids this sensitivity by using the whole profile rather than a single summary metric for recovery. This noise is highlighted in the right-side plots. In these plots: the top shows countries/regions grouped and colored by K-means cluster; the bottom shows countries/regions grouped by tercile but still colored by K-means cluster. Related to STAR Methods.

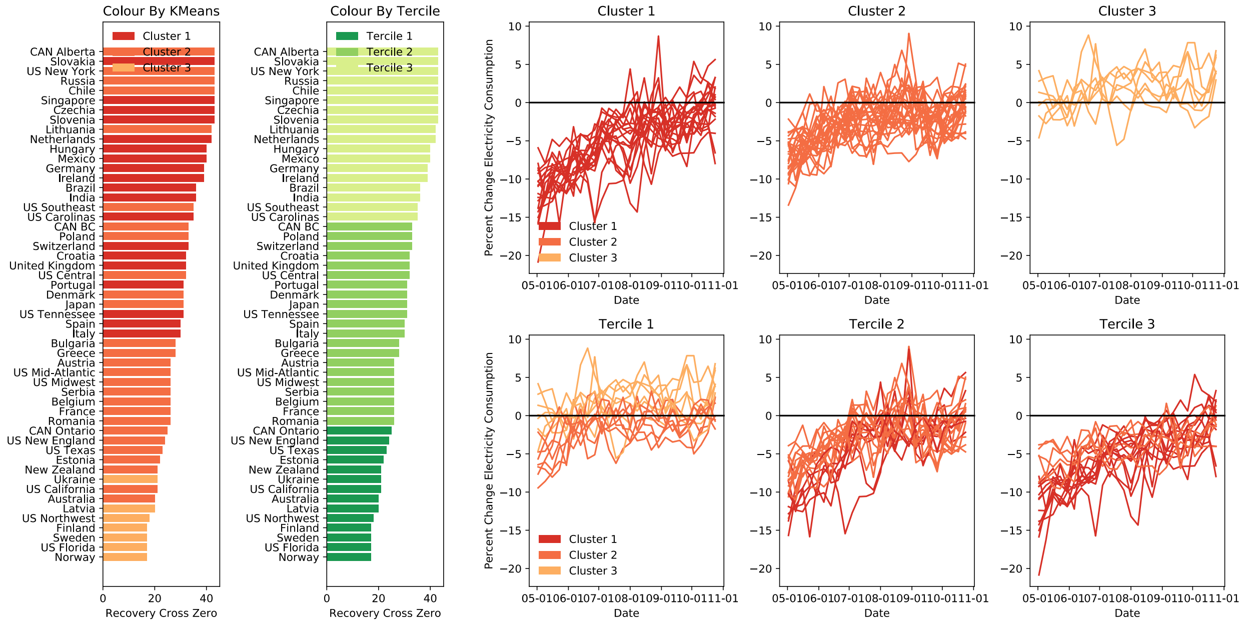

Figure S8: Comparison of K-means with tercile analysis in recovery period using mean value.

The mean value during the recovery period was used here to group countries into terciles. In the two left most plots this metric is plotted for all countries/regions; the coloring showing the K-means and tercile groupings reveal significant differences between the two. The K-means is able to identify the small cluster of “already recovered” countries with mean values greater than zero, but the tercile analysis includes these with many other countries still undergoing recovery. This is confirmed in the right timeseries plot. We also observe that several Cluster 1 (“Slow”) countries fall in Tercile 2, and vice versa, reflecting the sensitivity of the tercile method and the mean value metric to noise in the timeseries. Related to STAR Methods.

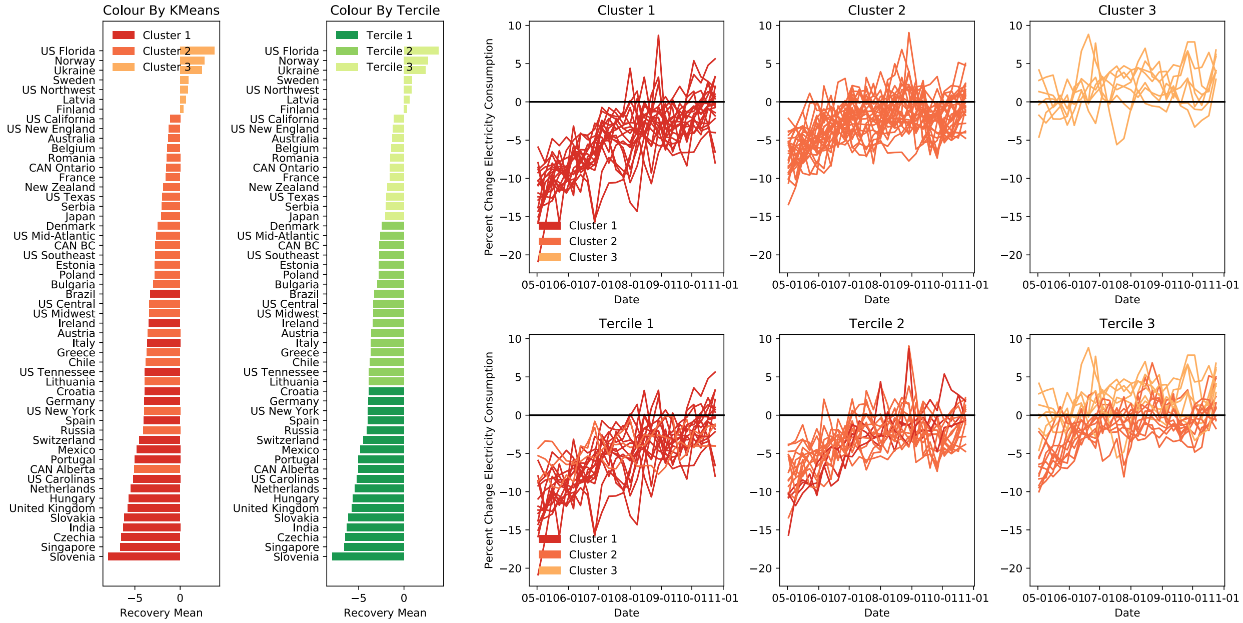

Figure S9: Comparison of K-means with tercile analysis in recovery period using total slope.

The total slope during the recovery period was used here to group countries into terciles, calculated as the difference between the first and last weeks in the percent change in electricity consumption for each country, divided by the number of weeks in the period. In the two left most plots this metric is plotted for all countries/regions, and on the right the countries/regions are grouped either by their cluster (top) or tercile (bottom). These reveal that the two approaches, K-means clustering of the recovery timeseries and tercile analysis of the total slope, capture very different features. The tercile approach groups countries more by the magnitude of their change through the recovery period than by their actual recovery. For example, tercile 3 includes countries with relatively flat profiles from all three clusters: some already recovered, some hovering below zero, and some in low plateaus. Related to STAR Methods.

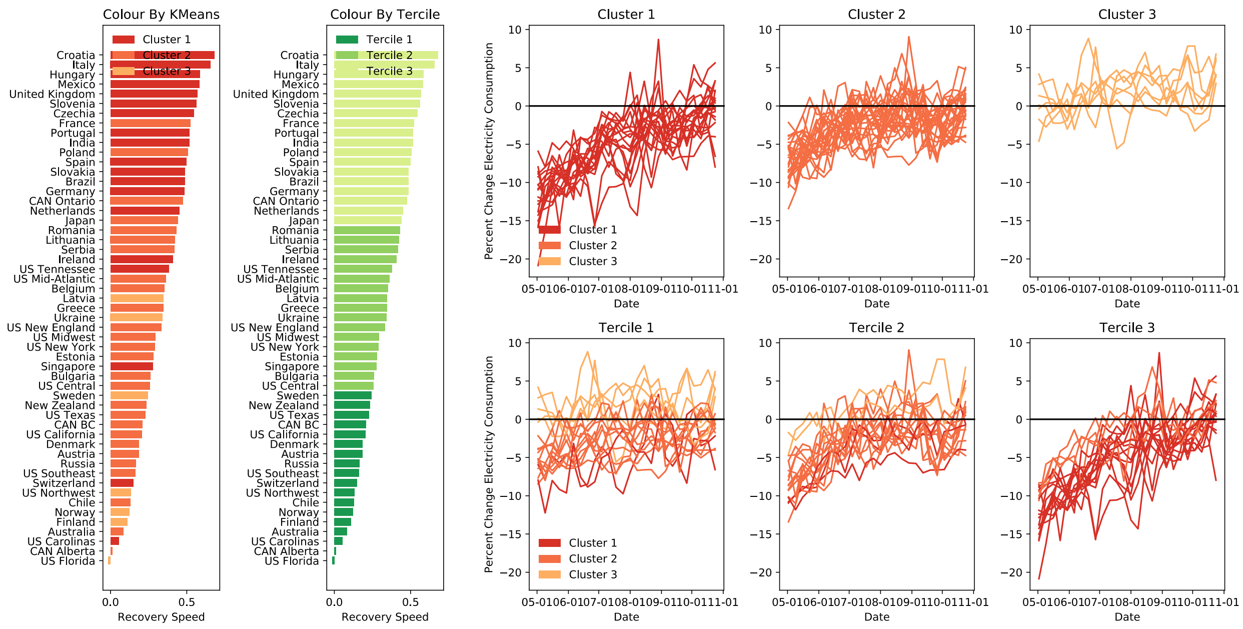

Figure S10: Relationships between electricity consumption impact groups and other country/region factors during the initial and recovery phases

Each bar gives the mean of the countries in the given cluster. For the health, stringency index, and mobility timeseries, the value for each country is its mean over the given period. Each error bar represents the standard error of the mean, calculated as the standard deviation divided by the square root of the number of observations. Related to STAR Methods.

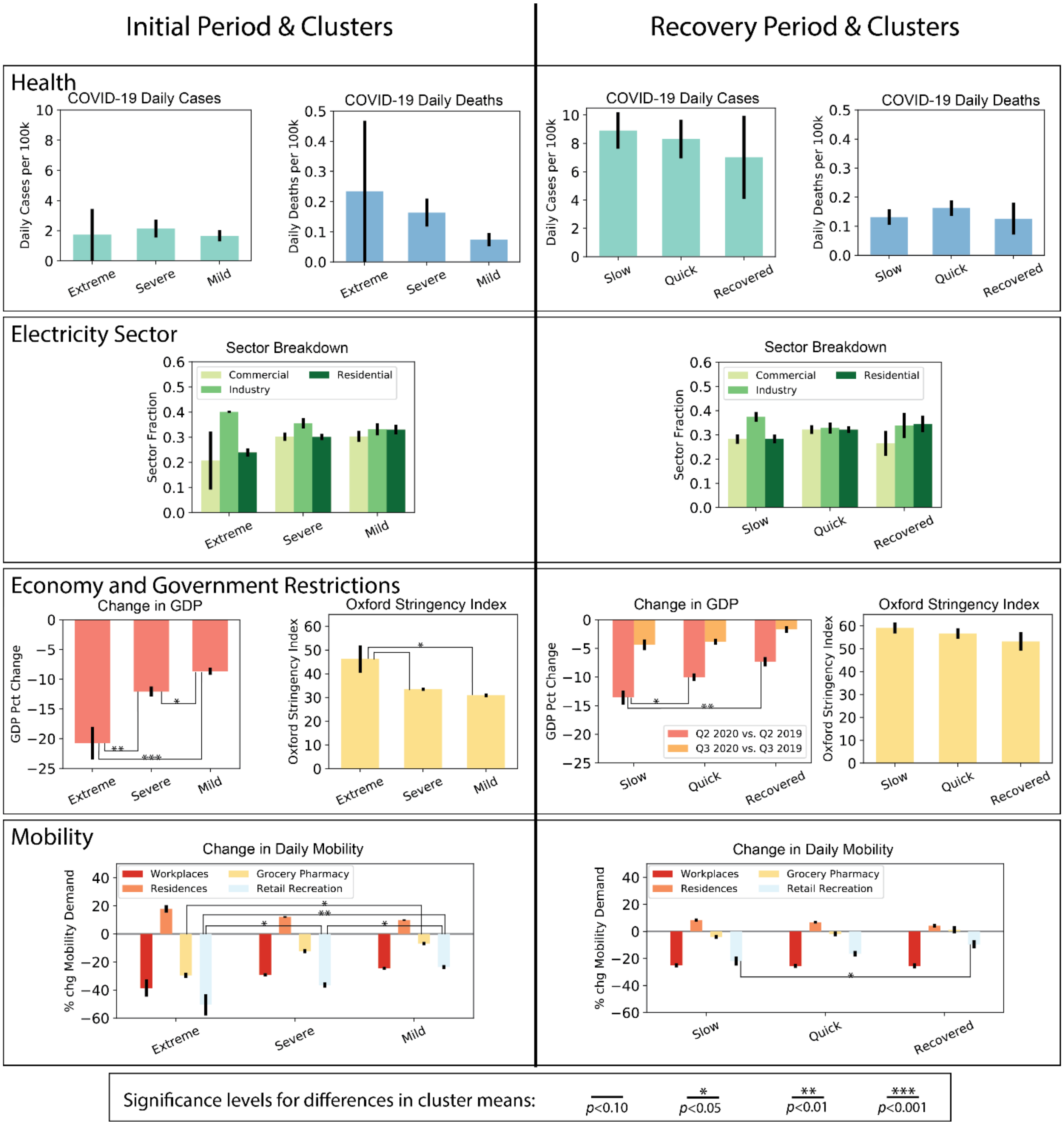

Table S1. One-way ANOVAs of cluster-level variation of additional variables.

Variable	Initial				Recovery			
	Extreme	Severe	Mild	p-value	Slow	Quick	Recovered	p-value
Oxford Stringency Index	46	33	31	.052[†]	59	57	53	.298
Mobility: Workplaces	-39	-29	-25	.380	-24	-26	-26	.934
Mobility: Residences	18	12	10	.344	8	7	4	.100
Mobility: Transit Stations	-47	-35	-29	.410	-27	-27	-23	.620
Mobility: Grocery and Pharmacy	-30	-12	-7	.049*	-3.5	-1.9	1.3	.172
Mobility: Retail and Recreation	-51	-36	-24	<.001***	-21	-17	-10	.018*
Mobility: Parks	-39	-7	6	.233	40	54	83	.169
Q2-2020 change in GDP (from Q2-2019)	-21	-12	-9	<.001***	-13	-10	-7	.003**
Q3-2020 change in GDP (from Q3-2019)	-6	-4	-3	.441	-4	-4	-2	.208
Sector: % Commercial	21	30	30	.413	28	32	26	.345
Sector: % Residential	24	30	33	.214	28	32	35	.098
Sector: % Industrial	40	36	34	.606	38	33	34	.362
Sector: % Transport	3	2	2	.757	2.0	1.7	1.6	.834
Sector: % Other	13	3	2	.006**	4.0	1.7	3.5	.276
Daily Cases per 100k	1.7	2.1	1.7	.359	8.2	7.7	6.5	.767
Daily Deaths per 100k	0.23	0.16	0.08	.795	0.13	0.16	0.12	.569

***p<.001; **p<.01; *p<.05, [†]p<.1

Table S2. Tukey HSD tests of pairwise cluster-level differences in additional variables.

Variable	Initial						Recovery					
	Cluster: Severe vs Extreme		Cluster: Mild vs Extreme		Mild vs Severe		Cluster: Quick vs Slow		Cluster: Recovered vs Slow		Recovered vs Quick	
	Mean diff	p-value	Mean diff	p-value	Mean diff	p-value	Mean diff	p-value	Mean diff	p-value	Mean diff	p-value
Oxford Stringency Index	-11.22	.064†	-12.26	.040*	-1.03	.847	-1.86	.649	-4.75	.276	-2.89	.586
Mobility: Workplaces	5.72	.346	5.23	.414	-0.48	.949	-0.44	.965	0.35	.989	0.79	.942
Mobility: Residences	-4.23	.357	-4.54	.311	-0.31	.962	-1.31	.544	-3.97	.083	-2.66	.281
Mobility: Transit Stations	7.59	.585	9.73	.422	2.14	.752	0.45	.989	4.43	.615	3.99	.648
Mobility: Grocery & Pharmacy	10.05	.104	12.02	.044*	1.97	.548	2.32	.501	5.62	.159	3.30	.488
Mobility: Retail & Recreation	17.05	.036*	23.73	.003**	6.69	.033*	5.18	.194	12.42	.016*	7.24	.192
Mobility: Parks	49.01	.267	54.30	.204	5.30	.900	11.57	.643	36.06	.145	24.49	.366
Q2-2020 change in GDP (from Q2-2019)	9.00	.004**	12.07	<.001***	3.07	.012*	3.13	.032*	5.80	.005**	2.67	.258
Q3-2020 change in GDP (from Q3-2019)	2.49	.534	2.95	.422	0.46	.866	0.25	.963	2.41	.203	2.16	.242
Sector: % Commercial	9.22	.390	9.19	.398	-0.03	1	3.38	.491	-1.66	.918	-5.04	.436
Sector: % Residential	5.80	.555	8.57	.289	2.77	.436	3.86	.233	6.68	.122	2.82	.656
Sector: % Industrial	-4.09	.862	-6.48	.696	-2.38	.732	-4.65	.349	-4.12	.662	0.52	.993
Sector: % Transport	-1.06	.762	-0.83	.847	0.23	.925	-0.36	.842	-0.40	.902	-0.04	.999
Sector: % Other	-9.87	.007**	-10.44	.004**	-0.57	.888	-2.24	.267	-0.50	.967	1.73	.652
Daily Cases per 100k	3.37	.505	2.09	.768	-1.28	.520	-0.10	.997	-1.29	.768	-1.19	.782
Daily Deaths per 100k	0.02	.974	0.00	.999	-0.02	.779	0.03	.717	-0.02	.937	-0.05	.629

***p<.001; **p<.01; *p<.05, †p<.1

Table S3. Results from panel regression analysis with region/country fixed effects for the initial and recovery period.

The dependent variable is daily percent change in electricity consumption. Related to Table 1.

	Model A Initial period[1]		Model B Recovery period	
Variable	Estimate[2]	*p*-value	Estimate[2]	*p*-value
Oxford Stringency Index	-0.0647***	<0.001	-0.0808***	<0.001
COVID-19 daily death rate (2-week average)	-1.6247***	<0.001	-0.2424	0.343
Change in daily mobility: retail and recreation	0.0688***	<0.001	0.0525***	<0.001

R-squared (projected model)[3]	0.496	0.094
R-squared (full model)[3]	0.653	0.348
Total sample size<br>Groups<br>Time intervals	3233<br>53<br>61	8760<br>53<br>124-169

[1] Due to data availability, the initial period ranged from February 15 to April 28.
[2] Significance levels: ***p<.001; **p<.01; *p<.05
[3] "Full model" refers to an R-squared calculated with fixed effects included. "Projected model" refers to an R-squared calculated for an estimated model where fixed effects are not included.

Table S4. Summary of methodology of relevant literature evaluating the impacts of COVID-19 on aggregate-level electricity demand or consumption.

This table summarizes the relevant literature evaluating the impacts of COVID-19 on aggregate-level electricity demand or consumption, and the types of analysis used in each paper. It does not include review papers that only cited other papers' modeling results and did not provide their own quantitative analysis. It also does not include papers that only focused on supply-side impacts.

The "Number of countries" refers to the number of unique countries considered in the analysis. Some papers also considered several subregions within each country, which are not included in this table.

For the demand/consumption modeling approach, "Historical comparison" means demand/consumption was directly compared to values from pre-pandemic data, either with or without corrections for temperature. The "Impact from model coefficients" approach means the impact of the pandemic was calculated directly from coefficients of a regression model. The "Baselining" approach means that a predictive model was used to estimate what electricity demand/consumption would have been during the COVID-19 time period with no pandemic.

An 'X' under "Variables accounted for in the demand/consumption estimate" means that the paper used some method to correct for temperature or temporal variables (e.g., day of week, seasonality, holidays). For temperature, this includes including temperature-related term(s) in a model or correcting for temperature when comparing to historical data. An 'X' under temporal variables only applies to approaches that explicitly modeled consumption/demand. If it was unclear from the paper whether these variables were accounted for in the methodology, then an 'X' was not included.

"Out-of-sample validation" applies primarily to papers that used the "Baselining" modeling approach.

Related to STAR Methods.

**Paper**	**Number of countries**	**COVID-19 analysis time period**		**Demand/consumption modeling approach**				**Variables accounted for in the demand/ consumption estimate**		**Out-of-sample validation**
		Start	*End*	*Historical comparison*	*Impact from model coefficients*	*Baselining*	*Other*	*Temperature*	*Temporal variables*	
This paper	45	Jan 2020	Oct 2020			X		X	X	X
McWilliams and Zachmann, 2020b (Bruegel electricity tracker)	35	Mar 2020	Jun 2021	X				X		
McWilliams and Zachmann, 2020a	30	Mar 2020	Apr 2020	X				X		
EPIC 2020 (EPIC COVID-19 website)	23	Jan 2020	May 2021		X*	X		X	X	
Cicala, 2020a	20	Feb 2020	Apr 2020		X			X	X	
Werth et al., 2020	16	Jan 2020	Jun 2020	X						
Lopez Prol and O, 2020	7	Mar 2020	Jul 2020			X		X	X	X
IEA, 2021	7	Jan 2020	Dec 2020	X*				X		
Bahmanyar et al·, 2020	6	Apr 2020	Apr 2020	X						
Narajewski and Ziel, 2020	5	Jan 2020	Apr 2020			X		X	X	
Halbrugge et al., 2021	5	Jan 2020	Jul 2020	X						
Percy and Mountain, 2020	4	Mar 2020	Apr 2020	X						
Hauser et al., 2021	3	Jan 2020	Aug 2020		X				X	
Carroll et al., 2020	2	Mar 2020	Apr 2020			X		X	X	
Cicala, 2020b	1	Jan 2020	Jul 2020		X			X	X	
Gillingham et al., 2020	1	Mar 2020	Jun 2020		X			X		
Ruan et al., 2020	1	Feb 2020	Jun 2020			X		X	X	X
Benatia, 2020	1	Mar 2020	Apr 2020			X		X	X	X
Leach et al., 2020	1	Jan 2020	Jun 2020		X			X	X	
Santiago et al., 2021	1	Feb 2020	Apr 2020	X						
Edomah and Ndulue, 2020	1	Mar 2020	Apr 2020	X						
Agdas and Barooah, 2020	1	Jan 2020	May 2020	X		X		X	X	X
Ghiani et al., 2020	1	Mar 2020	Apr 2020	X						
de Mello Delgado et al., 2021	1	Jan 2020	Sep 2020	X						
Liu and Lin, 2021	1	Apr 2020	Sep 2020				X	X		X
Kirli et al., 2021	1	Mar 2020	Mar 2020	X						
Alhajeri et al., 2020	1	Mar 2020	May 2020	X		X		X	X	
Alkhraijah et al., 2021	1	Jan 2020	Jun 2020				X	X		

* Estimate based on limited documentation

Table S5. Sources of electricity, weather, holiday, mobility, health, and GDP data.

Data type	Source	Identifier
United States sub-regional electricity data	US Energy Information Administration (EIA)	https://www.eia.gov/beta/electricity/gridmonitor/dashboard/electric_overview/US48/US48
European country-level electricity data	European Network of Transmission System Operators for Electricity (ENTSO-E)	https://transparency.entsoe.eu/
Australia electricity data	Australian Energy Market Operator (AEMO)	https://www.aemo.com.au/energy-systems/electricity/national-electricity-market-nem/data-nem/aggregated-data
Ontario, Canada electricity data	Independent Electricity System Operator (IESO)	http://www.ieso.ca/en/power-data
Alberta, Canada electricity data	Alberta Electric System Operator (AESO)	https://www.aeso.ca/market/market-and-system-reporting/data-requests
British Columbia, Canada electricity data	BC Hydro	https://www.bchydro.com/energy-in-bc/operations/transmission/transmission-system/balancing-authority-load-data.html
Japan electricity data	Tokyo Electric Power Company (TEPCO)	https://www.tepco.co.jp/en/forecast/html/download-e.html
New Zealand electricity data	New Zealand Electricity Authority	https://www.emi.ea.govt.nz/Wholesale/Reports/W_GD_C
Russia electricity data	SO-CDU	http://www.so-cdu.ru/index.php?id=972&tx_ms1cdu_pi1
Mexico electricity data	Mexico Centro Nacional de Control de Energía (CENACE)	https://www.cenace.gob.mx/Paginas/SIM/Reportes/EstimacionDemandaReal.aspx
Brazil electricity data	Brazil Operador Nacional do Sistema Elétrico (ONS)	http://www.ons.org.br/Paginas/resultados-da-operacao/historico-da-operacao/curva_carga_horaria.aspx
Chile electricity data	Chile Coordinador Eléctrico Nacional (CEN)	https://www.coordinador.cl/operacion/graficos/demanda/demanda-real-demanda/
India electricity data	India Power System Operation Corporation Limited (POSOCO)	https://posoco.in/reports/daily-reports
Singapore electricity data	Singapore Energy Market Authority (EMA)	https://www.ema.gov.sg/TemStatistic.aspx?pagesid=20140926wbNYp2Yh8iqy&pagemode=live&sta_sid=20140826Y84sgBebjwKV
China electricity data	China Electricity Council	http://www.cec.org.cn/menu
South Africa electricity data	Open Data for Africa	https://southafrica.opendataforafrica.org/wsblplg/electricity-generated-and-available-for-distribution-of-south-africa-monthly-update
Thailand electricity data	Thailand Energy Policy and Planning Office (EPPO)	http://www.eppo.go.th/index.php/en/en-energystatistics/electricity-statistic
Argentina electricity data	Argentina CAMMESA	https://portalweb.cammesa.com/Memnet1/default.aspx
Kenya electricity data	Kenya National Bureau of Statistics (KNBS)	https://www.knbs.or.ke/?wpdmpro=leading-economic-indicators-may-2020
US heating and cooling degree days	National Oceanic and Atmospheric Administration (NOAA)	https://www.cpc.ncep.noaa.gov/products/analysis_monitoring/cdus/degree_days/

Temperature measurements from ASOS weather system network	Iowa State University (ISU)	https://mesonet.agron.iastate.edu/request/download.phtml
Public holidays and observances	Time and Date website	https://www.timeanddate.com/holidays/
Change in human mobility patterns	Google Community Mobility Report	https://www.google.com/covid19/mobility/
Daily COVID-19 cases and deaths	Johns Hopkins University	https://coronavirus.jhu.edu/
Electricity sector breakdowns	International Energy Agency (IEA)	https://www.iea.org/data-and-statistics?country=USA&fuel=Electricity%20and%20heat&indicator=ElecConsBySector
US quarterly real GDP for 2020 Q2	US Bureau of Economic Analysis (BEA)	https://www.bea.gov/news/2020/gross-domestic-product-state-2nd-quarter-2020
US quarterly real GDP for 2020 Q3	US Bureau of Economic Analysis (BEA)	https://www.bea.gov/news/2020/gross-domestic-product-state-3rd-quarter-2020
Quarterly real GDP for most countries	OECD	https://stats.oecd.org/index.aspx?queryid=350
Singapore quarterly real GDP for 2020 Q2	News article (Business Times)	https://www.businesstimes.com.sg/government-economy/singapore-q2-gdp-plunges-by-132-in-worst-quarter-on-record
Singapore quarterly real GDP for 2020 Q3	News article (Focus Economics)	https://www.focus-economics.com/countries/singapore/news/gdp/revised-estimate-reveals-softer-gdp-contraction-in-q3
Croatia quarterly real GDP 2020 Q2	News article (Total Croatia News)	https://www.total-croatia-news.com/business/46150-croatia-s-q2-gdp-contracts-by-record-high-15
Croatia quarterly real GDP 2020 Q3	News article (Focus Economics)	https://www.focus-economics.com/countries/croatia/news/gdp/gdp-contracts-at-softer-albeit-still-pronounced-pace-in-q3
Serbia quarterly real GDP 2020 Q2	News article (Budapest Business Journal)	https://bbj.hu/economy/statistics/figures/serbia%CA%BCs-gdp-declines-in-q2
Serbia quarterly real GDP 2020 Q3	News article (SeeNews)	https://seenews.com/news/serbias-gdp-shrinks-14-yy-in-q3-stats-office-722727
Czech Republic quarterly real GDP 2020 Q2	News article (Focus Economics)	https://www.focus-economics.com/countries/czech-republic/news/gdp/gdp-records-largest-drop-on-record-in-q2-amid-covid-19-health
Czech Republic quarterly real GDP 2020 Q3	News article (Focus Economics)	https://www.focus-economics.com/countries/czech-republic/news/gdp/gdp-rebounds-in-q3-on-easing-restrictions
Ukraine quarterly real GDP 2020 Q2	News article (Focus Economics)	https://www.focus-economics.com/countries/ukraine/news/gdp/gdp-contracts-at-fastest-pace-in-over-four-years-in-q2
Ukraine quarterly real GDP 2020 Q3	News article (Focus Economics)	https://www.focus-economics.com/countries/ukraine/news/gdp/second-estimate-confirms-softer-contraction-in-gdp-in-q3
Oxford Stringency Index	Oxford COVID-19 Government Response Tracker	https://www.bsg.ox.ac.uk/research/research-projects/coronavirus-government-response-tracker
Population by country/state/province	World Bank	https://data.worldbank.org/indicator/SP.POP.TOTL
Population by country/state/province	Statistics Canada	https://www.statcan.gc.ca/
